# Circadian Phase Determines Tissue-Specific Adaptations to Long-Term Exercise in Obese Mice

**DOI:** 10.3390/nu17203281

**Published:** 2025-10-18

**Authors:** Shuo Wang, Ziwei Zhang, Jiapeng Huang, Yishan Tong, Cong Wu, Haruki Kobori, Sihui Ma, Katsuhiko Suzuki

**Affiliations:** 1 Graduate School of Sport Sciences, Waseda University, Tokorozawa 359-1192, Japan; 2 Faculty of Sport Sciences, Waseda University, Tokorozawa 359-1192, Japan

**Keywords:** circadian rhythm, exercise timing, chrono-exercise, high-fat diet, triglycerides, hepatic steatosis, adipose lipolysis

## Abstract

**Background**: Exercise interacts closely with the circadian system; however, whether long-term training elicits time-of-day-dependent metabolic adaptations in the context of obesity remains unclear. **Methods**: Male C57BL/6 mice were fed a high-fat diet and trained on a treadmill for 8 weeks during either the early rest phase (ZT3, Zeitgeber time) or the early active phase (ZT15). Sedentary mice served as controls. After the last session, animals were fasted for 4 h and sampled 48–49 h later. Plasma triglycerides (TGs) and glucose, as well as liver and epididymal white adipose tissue (EPI), were analyzed. **Results**: Plasma TGs showed a significant phase × exercise interaction (F(1, 25) = 5.25, *p* = 0.0307), with the lowest levels in ZT15-exe (27.22 mg/dL) compared with ZT15-sed (39.47 mg/dL, *p* < 0.01) and ZT3-exe (41.80 mg/dL, *p* < 0.01). Hepatic TG content was markedly lower in ZT3 than in ZT15 mice (F(1, 25) = 15.49, *p* < 0.001), and Oil Red O staining was associated with reduced lipid accumulation in exercised groups (*p* < 0.05). In EPI, *Fasn* expression was robustly decreased by exercise (F(1, 25) = 16.43, *p* = 0.0004, q = 0.0059), indicating long-term suppression of lipogenesis. In the liver, *Cpt1a* showed significant main effects of both phase (F(1, 25) = 10.11, *p* = 0.0039, q = 0.0158) and exercise (F(1, 25) = 13.42, *p* = 0.0012, q = 0.0353), being higher in ZT3 and under sedentary conditions, suggesting a circadian-dominant oxidative advantage in hepatic metabolism. **Conclusions**: Long-term exercise induced phase-dependent adaptations in lipid metabolism. Active-phase exercise promoted adipose lipid mobilization and lowered plasma TGs, while rest-phase training enhanced hepatic oxidative capacity. These results suggest a “tissue × time” framework of circadian-specific exercise responses, providing hypothesis-generating evidence for optimizing exercise timing in metabolic disorders.

## 1. Introduction

### 1.1. Research Background and Theoretical Foundation

The global escalation of obesity continues to drive the burden of type 2 diabetes, cardiovascular disease, and metabolic dysfunction-associated steatotic liver disease (MASLD; formerly known as non-alcoholic fatty liver disease, NAFLD) [1,2,3,4]. Meta-analytic estimates place the prevalence of MASLD near 25% worldwide, with substantially higher rates among individuals with obesity or diabetes [4]. Population-based cohorts also reveal marked race- and sex-specific differences in obesity-related vascular risk [3]. While positive energy balance and sedentary behavior remain proximal drivers of weight gain [5,6], structured exercise is an effective, low-risk intervention that improves multiple domains of metabolic health and cardiorespiratory fitness [7,8]. Beyond energy balance per se, mounting evidence indicates that circadian disruption is an important, modifiable contributor to metabolic disease.

The circadian timing system comprises a central pacemaker in the suprachiasmatic nucleus and peripheral clocks across metabolic organs, collectively orchestrating behavior, hormone secretion, and substrate metabolism [9,10,11]. Disruption of this system increases vulnerability to metabolic diseases with inflammatory features, such as obesity and diabetes [12], while endocrine mediators including adiponectin exhibit circadian oscillations that are metabolically relevant [11]. Nutritional challenges, particularly a high-fat diet (HFD), dampen circadian amplitude and shift the phase of clock-controlled genes in peripheral tissues, leading to behavioral rhythm disturbances and impaired metabolic flexibility [13,14,15]. At the molecular level, HFD-induced reprogramming of the hepatic transcriptome further disrupts the normal diurnal balance between lipid synthesis and oxidation [16]. Given this tight interconnection between circadian regulation and metabolism, exercise has emerged as a potent nonphotic zeitgeber capable of modulating both central and peripheral clocks. Appropriately timed exercise can shift the phase of core clock gene expression and thereby contribute to the realignment of circadian metabolic rhythms [17]. Recent reviews further highlight that circadian-aligned behaviors—particularly the timing of exercise (chrono-exercise) and feeding (chrono-nutrition)—jointly regulate metabolic flexibility, insulin sensitivity, and mitochondrial function [18]. Taken together, these findings highlight exercise as a promising behavioral strategy to restore or re-entrain circadian metabolic rhythms.

### 1.2. Previous Research and Evidence

Recent acute studies have revealed distinct time-of-day-dependent metabolic signatures following a single bout of exercise [19]. In adipose tissue, early active-phase exercise (e.g., ZT15, Zeitgeber time) preferentially induces genes for lipolysis and thermogenesis, whereas responses are attenuated in the early rest phase (ZT3) [20]. Notably, obesity diminishes this phase dependency, suggesting reduced metabolic plasticity under HFD conditions [21]. Collectively, acute studies indicate phase gating of exercise responses, but whether such gating persists after long-term training remains insufficiently defined.

Studies of long-term exercise interventions have likewise revealed pronounced time-of-day-dependent differences. Voluntary wheel running over several weeks was shown to alter clock gene expression, with phase advancement in the liver and adipose tissue and markedly increased expression levels in skeletal muscle [22]. Another study demonstrated that maximal running speed and endurance were enhanced in the late active phase, whereas no improvement was observed in the early active phase [23]. Forced treadmill training further supported these differences: endurance capacity was significantly improved when training occurred in the late active phase but was less effective in the early active phase, a discrepancy associated with liver glycogen content and feeding behavior [24]. In obesity models, comparison of early versus late active-phase exercise revealed that only late-phase training significantly ameliorated MASLD, whereas early-phase training was ineffective [25]. Collectively, these findings underscore the critical role of exercise timing in shaping long-term adaptations.

### 1.3. Research Gaps and Objectives

Despite growing evidence for the importance of exercise timing, several research gaps remain. First, most studies have focused on acute or short-term interventions [20,22], with limited verification of long-term adaptations. Although a two-week voluntary wheel-running study [22] showed that skeletal muscle rhythmicity and exercise performance peaked during the late active phase; however, voluntary activity differs fundamentally from forced treadmill exercise in both physiological load and experimental control. Furthermore, previous studies—whether voluntary or forced—have rarely examined exercise performed during the rest phase [23]. Thus, systematic comparisons of long-term exercise between the rest phase (ZT3) and the active phase (ZT15) in HFD-induced obese models remain scarce. Second, the synergistic mechanisms between the liver and adipose tissue that underlie homeostatic adaptations to exercise performed at different circadian phases remain poorly understood. Beyond single-tissue outcomes, the coordination between hepatic lipid handling and adipose mobilization represents a critical yet unresolved component of circadian exercise adaptation. Together, these gaps highlight the need to clarify how circadian timing governs tissue-specific metabolic adaptations to long-term exercise in obesity.

To address these questions, we employed an HFD-induced obese mouse model under controlled dietary and light–dark conditions, allow detailed molecular analysis of liver and adipose tissue responses under standardized experimental settings. We hypothesized that long-term exercise would induce tissue-specific adaptations governed by circadian phase, with active-phase training (ZT15) favoring lipid mobilization in adipose tissue and rest-phase training (ZT3) promoting a hepatic oxidative advantage. The results demonstrate that long-term exercise training produces distinct, tissue-specific adaptations governed by circadian phase, underscoring chrono-exercise as a critical design variable for metabolic interventions in obesity.

## 2. Materials and Methods

### 2.1. Experimental Approach

This controlled experimental study used seven-week-old healthy male C57BL/6J mice (*n* = 32), obtained from Takasugi Experimental Animals Supply Co., Ltd. (Kasukabe, Japan). All experimental procedures were approved by the Institutional Animal Care and Use Committee of Waseda University (Saitama, Japan; approval number: A23-126, approved on 11 April 2023) and conducted in accordance with the ARRIVE 2.0 guidelines.

The experiment comprised 8 weeks of HFD feeding followed by 8 weeks of treadmill training or sedentary control. Tissue collection was performed 48 h after the final exercise session following a 4 h fast, to minimize the acute effects of exercise. After euthanasia, tissues were rinsed with posphate-buffered saline (PBS) to remove surface blood, blotted dry, weighed, and immediately frozen in liquid nitrogen. All samples were stored at −80 °C until subsequent biochemical, histological, and molecular analyses. The prespecified primary endpoint was plasma triglycerides (TGs), and additional analyses of liver and adipose tissues were conducted to evaluate tissue-specific adaptations to exercise timing.

### 2.2. Animals and Circadian Phase Manipulation

Mice were housed in plastic cages (2–3 per cage) under controlled temperature and maintained on a 12:12 h light–dark cycle (lights on at 08:00 and off at 20:00), with ZT 0 defined as lights on and ZT12 as lights off. Standard bedding was provided throughout the experiment and replaced weekly. To minimize baseline differences in body weight, mice were assigned to two groups using pairwise randomization based on body weight. After a 2-week acclimatization period, all mice were ranked by body weight, and the overall mean was calculated. Animals with similar body weights around the mean were sequentially paired, and one mouse from each pair was alternately assigned to the ZT3 or ZT15 group. All mice had ad libitum access to an HFD (Research Diets D12492, E.P. Trading Co., Ltd., Tokyo, Japan; protein 20 kcal%, carbohydrate 20 kcal%, and fat 60 kcal%), and water.

**Figure 1:**
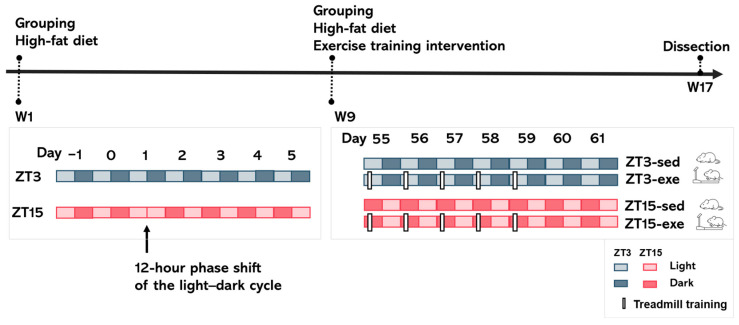
Experimental design. Male C57BL/6J mice were acclimated for 2 weeks under a 12:12 h light–dark cycle (lights on at ZT0 = 08:00) and then fed a high-fat diet (HFD; 60% kcal from fat) for 8 weeks. Mice were first assigned by pairwise randomization to two phase groups: ZT3 (*n* = 16; rest phase, blue), maintained on the standard light–dark cycle, and ZT15 (*n* = 16; active phase, red), which on Day 3 of HFD feeding underwent 24 h of constant light followed by a 12 h reversal (new ZT0 = 20:00). Each phase group was then subdivided into sedentary and exercise subgroups: ZT3-sed (*n* = 8), ZT3-exe (*n* = 8), ZT15-sed (*n* = 8), and ZT15-exe (*n* = 8). Exercise mice underwent treadmill training 5 sessions per week for 8 weeks. Forty-eight hours after the final session, mice were fasted for 4 h and euthanized for tissue collection to minimize acute exercise effects. ZT, Zeitgeber time; Sed, sedentary; Exe, exercise.

Two circadian phase groups were established (Figure 1):

ZT3 group (*n* = 16): Maintained under the standard light–dark cycle (ZT0 = 08:00).

ZT15 group (*n* = 16): On Day 3 of HFD feeding, mice were exposed to constant light for 24 h and then subjected to a 12 h reversal of the light–dark cycle (new ZT0 = 20:00) to align exercise timing with the mice’s natural active phase, while ensuring that training for the ZT3 and ZT15 groups was conducted at the same clock time and under identical environmental conditions. This procedure produced a stable 12 h phase delay while preserving a 24 h rhythm [26,27].

After 8 weeks of HFD feeding, each group was further subdivided into sedentary (ZT3-sed, *n* = 8, ZT15-sed, *n* = 8) and exercise (ZT3-exe, *n* = 8, ZT15-exe, *n* = 8) groups based on body weight (Figure 1). Sample size was determined based on previous similar studies and logistical feasibility [20,28]. Subgroup allocation was performed using the same pairwise randomization method described above to minimize baseline differences. During the entire intervention, body weight, food intake, and water intake were measured weekly, and feeding behavior was monitored 3–7 times per week at ZT0 (morning) and ZT12 (evening) to assess circadian rhythmicity. Food intake was recorded per cage (2–3 mice each) and averaged within each cage to obtain a single data point. Therefore, the statistical unit for feeding analyses was the cage (*n* = 6 cages per group during the HFD period and *n* = 3 cages per group during the exercise intervention).

Quality control and exclusion criteria: all histological and molecular analyses were conducted by investigators blinded to group allocation. A few animals were excluded from the final dataset due to unexpected death or severe fighting-related injuries. Predefined exclusion criteria included >15% body weight loss or visible injury, in accordance with institutional welfare policies. After exclusion, the final sample sizes ranged from *n* = 5 to 8 per group (Figure 1), ensuring data integrity and comparability.

### 2.3. Exercise Intervention Protocol

Exercise training began in Week 9 of HFD feeding and continued for 8 weeks (5 days/week, 60 min/day). Before the formal intervention, mice underwent a two-day familiarization period (10 and 12 m/min, 10 min/day) to reduce stress and ensure adaptation to treadmill running.

Exercise groups (ZT3-exe, ZT15-exe) performed treadmill running at their assigned circadian phase:

ZT3 (early rest phase): 11:00 local time.

ZT15 (early active phase): 23:00 local time after phase reversal.

Training was performed on a motorized treadmill (KN-73, Natsume Seisakusho Co., Ltd., Tokyo, Japan) with a 0° incline, following a progressive moderate-to-vigorous aerobic protocol corresponding to approximately 60–70% of VO_2_ max for obese C57BL/6J mice [29,30]. During exercise, if necessary, mice were gently encouraged to continue running by light tail tapping, and no electric shocks were applied. To minimize stress, the same operator manually adjusted the speed using a rotary dial at a steady pace, ensuring smooth acceleration while continuously monitoring the animals’ condition. The treadmill speed was set as follows: training started at 9 m/min in Week 9, increasing by 2 m/min every 10 min until the 20th minute, after which the speed was maintained for the remainder of the 60 min session. The initial speed increased by 1 m/min each subsequent week. By Week 13, the starting speed reached 13 m/min, rising to 16 m/min at 5 min and 17.5 m/min at 10 min, which was maintained thereafter. Training from Weeks 14–16 followed the same speed pattern. Sedentary controls (ZT3-sed and ZT15-sed) did not perform treadmill running but were placed around the treadmill at the same time to control for environmental factors.

At the end of the intervention, tissue sampling began 48 h after the final exercise session to avoid acute exercise effects. Prior to sampling, a 4 h fasting period was implemented to minimize the impact of food intake on metabolic parameters. Sampling and weighing were performed alternately between groups, with each tissue collected by the same investigator. Both the operator and the recorder of tissue weights were blinded to group allocation.

### 2.4. Blood Glucose Measurement

During the experiment, blood glucose levels were monitored at three time points: before HFD feeding (Week 0), before training (Week 8), and after training (Week 16). Blood glucose was measured using a glucometer (Free Style Precision Neo; Abbott Japan Co., Ltd., Tokyo, Japan) in combination with compatible electrodes (FS Precision Glucose Test Electrodes; Abbott Japan Co., Ltd., Tokyo, Japan). All measurements were conducted by the same operator, beginning at ZT2 for the ZT3 group and ZT14 for the ZT15 group, with alternate testing order to account for circadian influences. For each measurement, 1–2 μL of capillary whole blood was collected from the tail tip and applied to the electrode, and glucose concentration was automatically determined by the device via an electrochemical method.

### 2.5. Hepatic Lipid Extraction and TG Measurement

Hepatic lipids were extracted using a modified Bligh and Dyer chloroform–methanol method. Briefly, approximately 100 mg of frozen liver tissue was placed into a pre-weighed 2 mL microtube, and the net weight was recorded. A 10-fold volume of distilled water was then added, and the tissue was homogenized. A 440 μL aliquot of the homogenate was transferred into a new 2 mL tube, to which 1100 μL of chloroform–methanol solution (1:2, *v*/*v*; Chloroform, Cat# C2432, Sigma-Aldrich Japan, Tokyo, Japan; Methanol, Cat# 19-2420-3, Sigma-Aldrich Japan, Tokyo, Japan) was added. After thorough vortexing and centrifugation, the lower organic phase (chloroform layer) was collected. The solvent was evaporated at 80 °C until only lipid residues remained. The lipid residue was dissolved in 100 μL isopropanol (FUJIFILM Wako Pure Chemical Corporation, Osaka, Japan). The investigator performing liver sampling and TG assays was blinded to group allocation throughout the experiment. TG levels in the liver tissue were quantified using a commercial kit (FUJIFILM Wako Pure Chemical Corporation, Osaka, Japan) by colorimetric assay in a 96-well plate by a plate reader (SpectraMax iD5, Molecular Devices, San Jose, CA, USA). Assay reliability was verified by the standard calibration curve (R^2^ > 0.99) included in each batch. Concentrations were calculated from the calibration curve and expressed as milligrams of TGs per gram of wet tissue weight [31,32,33].

### 2.6. Assessment of Plasma Biochemical Markers

Blood samples were collected from the posterior vena cava of mice under anesthesia, and plasma was separated by centrifugation. All sampling and measurements were performed by the same operator, with alternating group order to minimize potential effects of sampling time differences between groups on the results. Plasma levels of non-esterified fatty acids (NEFAs), glucose, and TGs were measured in duplicate using commercial biochemical assay kits (FUJIFILM Wako Pure Chemical Corporation, Osaka, Japan) with a colorimetric method in 96-well plates and absorbance was determined using a microplate reader. Concentrations were calculated based on standard calibration curves.

Additional plasma biochemical parameters were analyzed by Kotobiken Medical Laboratories, Inc. (Tokyo, Japan), including liver function markers [aspartate aminotransferase (AST), alanine aminotransferase (ALT), and albumin]; lipid metabolism markers [total cholesterol (T-Cho), low-density lipoprotein cholesterol (LDL-C), and high-density lipoprotein cholesterol (HDL-C)]; myocardial and tissue damage markers [creatine kinase (CK) and lactate dehydrogenase (LDH)]; pancreatic function markers [amylase and lipase]; and renal function markers [blood urea nitrogen (BUN), creatinine (Cr), and uric acid (UA)]. These measurements provided a comprehensive evaluation of systemic metabolic status.

### 2.7. Oil Red O Staining

Frozen liver sections were air-dried at room temperature for 30 min, fixed in 10% neutral buffered formalin solution (Cat# 062-01661, FUJIFILM Wako Pure Chemical Corporation, Osaka, Japan) for 10 min, and then briefly rinsed with 60% isopropanol. The sections were subsequently stained in working Oil Red O solution (Cat# 4049-1, Muto Pure Chemicals Co., Ltd., Tokyo, Japan) for 15 min, followed by quick washes with 60% isopropanol and distilled water. Counterstaining was performed with Mayer’s hematoxylin (Cat# 131-09665, FUJIFILM Wako Pure Chemical Corporation, Osaka, Japan) for 1 min, rinsed with distilled water, and mounted with an aqueous mounting medium. Sections were photographed using an all-in-one fluorescence microscope (BZ-X800, KEYENCE, Osaka, Japan) equipped with a Plan Apo 20× objective lens (KEYENCE, Osaka, Japan).

For each mouse, 4–6 sections from different depths were analyzed. Five to six mice per group were selected for quantification to ensure representative morphology. Mice with extreme body weights or tissue abnormalities (e.g., excessive steatosis, damage) were excluded. A blinded investigator randomly selected ten non-overlapping fields per mouse, and ImageJ software (version 1.54f, National Institutes of Health, Bethesda, MD, USA) was used for quantification. The Oil Red O-positive area was expressed as a percentage of total tissue area (mean ± SEM, *n* = 5–6 per group; SEM = Standard error of the mean).

### 2.8. Quantitative Real-Time PCR (qPCR)

Total RNA was extracted using the NucleoSpin RNA Kit (Takara Bio Inc., Kusatsu, Japan) according to the manufacturer’s instructions, and RNA concentration and purity were determined using a spectrophotometer (NanoDrop Technologies, Wilmington, DE, USA). cDNA was synthesized using the PrimeScript^TM^ FAST RT Reagent Kit with gDNA Erase (Takara Bio Inc., Kusatsu, Japan). Quantitative PCR amplification was performed on a Thermal Cycler Dice^®^ Real Time System IV (Takara Bio Inc., Kusatsu, Japan) using TB Green^®^ Premix Ex Taq™ II FAST qPCR (Takara Bio Inc., Kusatsu, Japan), following the recommended protocol. To ensure accurate normalization, several candidate reference genes were initially evaluated for expression stability across groups. Among these, ribosomal protein S18 (*Rps18*) in liver and glyceraldehyde-3-phosphate dehydrogenase (*Gapdh*) in adipose tissue showed the lowest intra-group variability (coefficient of variation of Ct values < 2%) and were therefore selected as stable reference genes. Relative mRNA expression levels were calculated using the 2^–∆∆Ct^ method. Primer sequences for all target genes are listed in Table 1.

### 2.9. Statistical Analysis

Statistical analyses were performed using GraphPad Prism 10.4.0 (GraphPad Software, La Jolla, CA, USA). Data normality was tested with the Shapiro–Wilk test, and variance homogeneity with the F test (two groups) or Brown–Forsythe test (fore groups). When assumptions were violated, data were log-transformed or analyzed using nonparametric tests. Two-group comparisons used unpaired Student’s *t*-tests (equal variances) or Welch’s *t*-tests (unequal variances). Four-group comparisons applied two-way analysis of covariance (ANOVA, phase × exercise) to test main and interaction effects. Significant interactions were followed by simple main effect analyses with Bonferroni post hoc corrections. To control false discovery rate, *p* values were adjusted using the Benjamini–Hochberg FDR method, yielding both raw *p* and adjusted q values. Effect sizes (partial η^2^ for ANOVA; Hedges’ g for pairwise comparisons) and 95% confidence intervals (CIs) were reported. Significance was set at *p* < 0.05 (two-tailed). Data are presented as mean ± SEM, with 5–8 mice per group unless otherwise stated.

All analyses complied with ARRIVE 2.0 guidelines. In the main text, only statistically significant or biologically meaningful results are described, with nonsignificant findings summarized in the Supplementary Materials.

## 3. Results

### 3.1. Circadian and Metabolic Phenotypes During HFD and Exercise Intervention

#### 3.1.1. Circadian Regulation of Feeding Under HFD and Exercise Training

**Figure 2:**
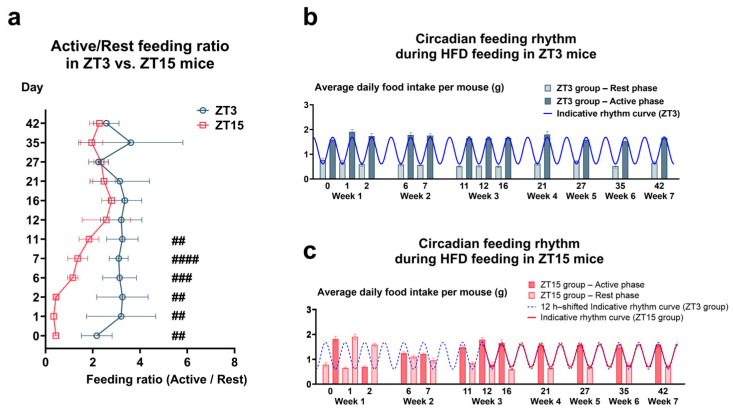
Circadian Feeding Rhythms under an HFD. (**a**) Active/rest feeding ratio in ZT3 vs. ZT15 mice. (**b**,**c**) Feeding rhythms under HFD: (**b**) ZT3 (normal light–dark cycle) and (**c**) ZT15 (12 h phase-shifted cycle). Each data point represents a cage-level mean (*n* = 6 cages per group), and data are presented as means ± standard error of the mean (SEM). Statistical analysis was performed using Welch’s *t*-test. Significance levels: ## *p* < 0.01; ### *p* < 0.001; #### *p* < 0.0001.

To evaluate whether a 12 h phase shift altered circadian feeding under HFD, food intake was measured at ZT0 (rest onset) and ZT12 (activity onset). Feeding data were analyzed as cage-level averages (*n* = 6 per group). In the ZT3 group, mice maintained on a standard light–dark cycle exhibited a clear rhythm, with food intake during the active phase ~2.8-fold higher than during the rest phase (Active/Rest ratio [A/R] = 2.78; Figure 2a,b; Table S1). In the ZT15 group, entrainment required ~12 days after light–dark reversal. From day 12 onward, their A/R ratio (~2.3) became comparable to that of ZT3 mice (Figure 2a,c; Table S1), indicating restoration of stable feeding rhythm. These data suggest that the 12 h phase-shifted mice achieved a stable feeding rhythm comparable to controls.

**Figure 3:**
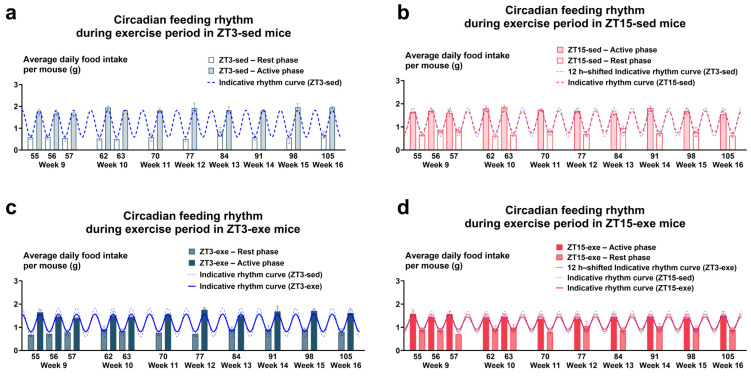
Circadian Feeding Rhythms during the Exercise Intervention. (**a**) ZT3-sed, (**b**) ZT15-sed, (**c**) ZT3-exe, and (**d**) ZT15-exe. Each data point represents a cage-level mean (*n* = 3 cages per group), and data are presented as means ± SEM. Statistical analysis was performed using two-way ANOVA (phase × exercise) followed by post hoc tests where appropriate. ANOVA, analysis of covariance.

During the 8-week intervention, feeding rhythms were again assessed by the A/R ratio. Sedentary ZT3 mice showed an A/R of 3.07, whereas sedentary ZT15 mice displayed a lower ratio of 2.27 (Figure 3a,b; Table S2). Exercise attenuated feeding rhythm amplitude in both phase groups: ZT3-exe showed a reduced A/R of 1.94, while ZT15-exe exhibited the lowest ratio (A/R = 1.60; Figure 3c,d). These results indicate that long-term treadmill exercise was associated with a reduction in feeding rhythm amplitude, particularly when performed during the early active phase (ZT15).

#### 3.1.2. Food and Water Intake and Body Weight Gain During HFD and Exercise Intervention

**Figure 4:**
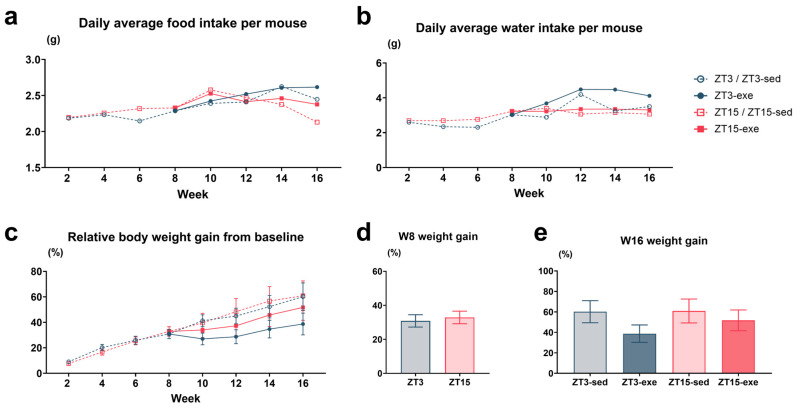
Feeding, drinking, and body weight changes during the exercise intervention. (**a**) Daily food intake, (**b**) daily water intake, (**c**) relative body weight gain (%) from week 0. (**d**) Body weight gain (%) at week 8. (**e**) Body weight gain (%) at week 16. Statistical analysis was performed using Welch’s *t*-test (two-group) or two-way ANOVA (phase × exercise; four-group) followed by post hoc tests where appropriate. Data are presented as mean ± SEM (ZT3 *n* = 13; ZT15 *n* = 16; ZT3-sed *n* = 5; ZT3-exe *n* = 8; ZT15-sed *n* = 8; ZT15-exe *n* = 8).

To evaluate the overall energy intake and the metabolic impact of our interventions, average daily food and water intake and body weight gain were systematically monitored throughout the study. The phase shift itself had minimal impact on total energy intake. During the initial 8-week HFD feeding period, the average daily food intake was comparable between the ZT3 (2.24 ± 0.03 g) and ZT15 (2.33 ± 0.03 g) groups. This pattern persisted during the subsequent 8-week exercise intervention, with no significant differences in food or water intake among the four groups (ZT3-sed: 2.45 ± 0.07 g; ZT3-exe: 2.51 ± 0.04 g; ZT15-sed: 2.43 ± 0.06 g; ZT15-exe: 2.43 ± 0.06 g) (Figure 4a,b).

Body weight increased progressively in all groups over the 16-week period (Figure 4c). At Week 8, before exercise, the weight gain in ZT15 mice (32.87% ± 4.10%) was slightly greater than in ZT3 mice (30.85% ± 3.63%), although this difference was not statistically significant by Welch’s *t*-test (Figure 4d). By the end of the intervention (Week 16), the mean body weight gain of sedentary controls (ZT3-sed: 60.22% ± 10.77%; ZT15-sed: 60.90% ± 11.66%) was numerically higher than that of exercised groups (ZT15-exe: 51.77% ± 10.15%; ZT3-exe: 38.73% ± 8.53%), and showed no significant main effects of phase or exercise according to two-way ANOVA (Figure 4e).

Overall, these results show that the phase shift and exercise interventions did not markedly affect energy intake, and changes in body-weight gain were not statistically significant.

#### 3.1.3. Effects of Exercise Timing on Relative and Absolute Tissue Weights

**Figure 5:**
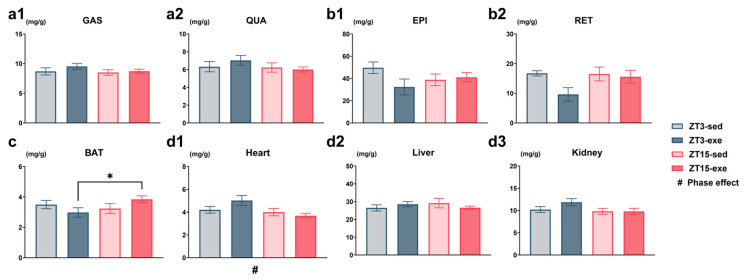
Relative tissue weights normalized to body weight in mice. (**a1**,**a2**) Skeletal muscle: gastrocnemius (GAS, (**a1**)), quadriceps (QUA, (**a2**)); (**b1**,**b2**) white adipose tissue (WAT): epididymal (EPI, (**b1**)), retroperitoneal (RET, (**b2**)); (**c**) brown adipose tissue (BAT); (**d1**,**d2**,**d3**) organs: heart (**d1**), liver (**d2**), kidney (**d3**). Statistical analysis was performed using two-way ANOVA (phase × exercise) followed by post hoc tests where appropriate. Data are presented as mean ± SEM (ZT3-sed *n* = 5; ZT3-exe *n* = 8; ZT15-sed *n* = 8; ZT15-exe *n* = 8). # Phase main effect; * post hoc comparison. Significance levels: #/* *p* < 0.05.

To determine the tissue-specific effects of exercise timing, we analyzed both relative (normalized to body weight) and absolute weights of key metabolic organs. All data were evaluated using two-way ANOVA (phase × exercise), followed by post hoc tests where applicable. The analysis of relative tissue weights (Figure 5) revealed several distinct patterns. A non-significant tendency toward an exercise effect was observed in retroperitoneal white adipose tissue (RET), with exercised groups showing lower mass (main effect of exercise, *p* = 0.0858; Figure 5(b2), Table S3). For brown adipose tissue (BAT), a phase × exercise interaction was observed at a marginal level (*p* = 0.0759), with the ZT3-exe group exhibiting significantly lower mass than the ZT15-exe group (*p* < 0.0397; Figure 5c, Table S3). A significant main effect of phase was found for the heart (*p* = 0.0357, Table S3), and the ZT3-sed group had a significantly lower relative heart mass than the ZT15-sed group (Figure 5(d1), Table S3).

Analysis of absolute tissue weights provided additional information (Figure S1). A significant interaction was observed for absolute heart weight (*p* = 0.0248) and post hoc analysis showed that ZT3-exe mice had a higher absolute heart weight than ZT15-exe mice (*p* = 0.0064; Figure S1(d1), Table S3). For the kidney, an interaction was suggestive but not statistically supported (*p* = 0.0886), with tendencies for ZT3-sed < ZT3-exe (*p* = 0.0904) and ZT3-exe > ZT15-exe (*p* = 0.0656; Figure S1(d3), Table S3).

Together, these results indicate that phase and exercise timing were associated with modest, tissue-dependent variations in organ mass, most notably in adipose tissue and the heart.

### 3.2. Primary Endpoint: Plasma TG and Other Metabolic Parameters

#### 3.2.1. Plasma TGs (Primary Endpoint), NEFAs, and Blood Glucose Concentrations After Exercise Intervention

**Figure 6:**
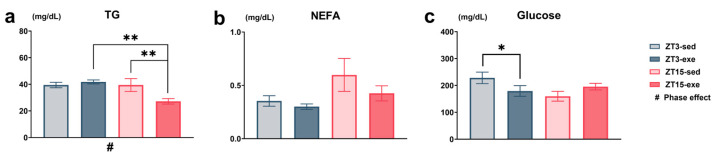
Plasma concentrations of triglycerides (TGs), non-esterified fatty acids (NEFAs), and glucose measured at the end of the intervention. (**a**) Plasma TGs (mg/dL); (**b**) plasma NEFAs (mg/dL); (**c**) plasma glucose (mg/dL). Statistical analysis was performed using two-way ANOVA (phase × exercise) followed by post hoc tests where appropriate. Data are presented as mean ± SEM (ZT3-sed *n* = 5; ZT3-exe *n* = 8; ZT15-sed *n* = 8; ZT15-exe *n* = 8). # Phase main effect; * post hoc comparison. Significance levels: #/* *p* < 0.05; ** *p* < 0.01.

To assess the combined effects of exercise timing and circadian phase on systemic metabolism, lipid concentrations and plasma glucose were measured after the intervention. All data were analyzed using two-way ANOVA (phase × exercise), followed by post hoc tests where appropriate. Plasma TGs, the prespecified primary endpoint, exhibited the most complex response among the measured plasma metabolites. Two-way ANOVA revealed significant main effects of phase (F(1, 25) = 5.25, *p* = 0.0306, partial η^2^ = 0.17) and a significant phase × exercise interaction (F(1, 25) = 5.25, *p* = 0.0307, partial η^2^ = 0.17). Post hoc analysis indicated that TG concentrations were significantly higher in ZT3-exe compared with ZT15-exe (mean difference = 14.58 mg/dL, t(25) = 3.47, 95% CI [5.94–23.23], *p* = 0.0019, Hedges’ g = 1.35), and higher in ZT15-sed compared with ZT15-exe (mean difference = 12.25 mg/dL, t(25) = 2.92, 95% CI [3.61–20.90], *p* = 0.0073, Hedges’ g = 1.13; Figure 6a). For plasma NEFAs, two-way ANOVA indicated a trend toward a main effect of phase (F(1, 25) = 3.47, *p* = 0.0742, partial η^2^ = 0.12), with the ZT3 groups exhibiting numerically lower concentrations compared to the ZT15 group (Figure 6b). Plasma glucose exhibited a significant phase × exercise interaction (F(1, 25) = 5.31, *p* = 0.0297, partial η^2^ = 0.18). Post hoc analysis revealed that ZT3-sed mice showed higher plasma glucose than ZT15-sed mice (mean difference = 68.33 mg/dL, t(25) = 2.48, *p* = 0.0202, Hedges’ g = 0.96, 95% CI [11.65, 125.10]; Figure 6c).

To further confirm that the differences in plasma metabolites were not influenced by baseline body weight, an analysis of covariance (ANCOVA) was performed using body weight at Week 2 (end of the adaptation period) and Week 8 (pre-exercise) as covariates (Table S4). The group effect on plasma TGs remained significant in both models (W2: F(3, 24) = 4.45, *p* = 0.0127; W8: F(3, 24) = 5.42, *p* = 0.0054), whereas the covariate (body weight) was not significant. Plasma glucose showed a similar trend after adjustment (W2, *p* = 0.0823; W8, *p* = 0.0664), and body weight was not significant in either model.

Overall, circadian phase remained the primary factor influencing plasma glucose levels, the effect on plasma NEFAs was limited, and the phase × exercise interaction primarily accounted for the observed variation in plasma TGs.

Collectively, these results reveal that circadian phase exerted a dominant influence on plasma glucose levels, while exercise was associated with a modest reduction in circulating NEFAs, and the phase × exercise interaction contributed to the observed variation in plasma TG levels.

#### 3.2.2. Circadian Variation in Tail-Tip Capillary Blood Glucose

**Figure 7:**
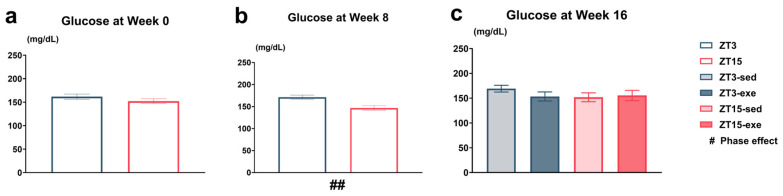
Temporal changes in tail-tip capillary blood glucose. (**a**) Glucose concentration at Week 0 (mg/dL); (**b**) glucose concentration at Week 8 (mg/dL); (**c**) glucose concentration at Week 16 (mg/dL). Statistical analysis was performed using Welch’s *t*-test (two-group) or two-way ANOVA (phase × exercise; four-group) followed by post hoc tests where appropriate. Data are presented as mean ± SEM (ZT3 *n* = 13; ZT15 *n* = 16; ZT3-sed *n* = 5; ZT3-exe *n* = 8; ZT15-sed *n* = 8; ZT15-exe *n* = 8). # Phase main effect. Significance levels: ## *p* < 0.01.

To investigate the circadian regulation of glucose homeostasis under a high-fat diet, blood glucose levels were measured at three time points using capillary whole blood collected from the tail tip. At Week 0, prior to HFD feeding and phase shift, blood glucose levels did not differ between the future ZT3 and ZT15 groups (Figure 7a). After 8 weeks of high-fat diet feeding (Week 8), a significant circadian difference was detected by Welch’s *t*-test, with glucose levels markedly higher in the ZT3 group compared to the ZT15 group (*p* = 0.0019; Figure 7b, Table S3). By the end of the intervention (Week 16), the average blood glucose level in ZT3-sed mice remained numerically higher than that in ZT15-sed mice, although the difference was not statistically significant (Figure 7c).

Overall, these findings indicate a temporary circadian variation in blood glucose under HFD conditions, with higher levels at ZT3 than at ZT15 during the middle phase of the experiment.

### 3.3. Effects of Exercise and Circadian Phase on Hepatic Lipid Accumulation and Plasma Biochemical Parameters

**Figure 8:**
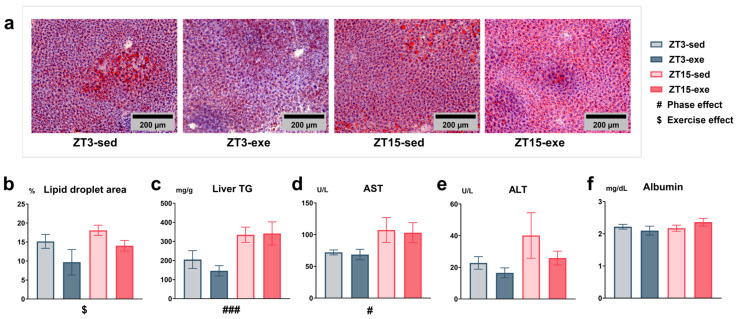
Effects of exercise and circadian phase on hepatic lipid accumulation and plasma liver function parameters. (**a**) Representative images of liver Oil Red O staining (captured with a 20× objective lens using a BZ-X800 microscope, KEYENCE, Japan; scale bar = 200 µm). Quantification was performed using ImageJ software on ten randomly selected non-overlapping fields per mouse, and the mean value was used for each animal (ZT3-sed *n* = 5; ZT3-exe *n* = 6; ZT15-sed *n* = 6; ZT15-exe *n* = 6). (**b**) Quantitative analysis of lipid droplet area (%Area). (**c**) Biochemical measurement of hepatic TGs (mg/g tissue). (**d**–**f**) Plasma liver function-related parameters: (**d**) aspartate aminotransferase (AST, U/L); (**e**) alanine aminotransferase (ALT, U/L); (**f**) albumin (g/dL). Statistical analysis was performed using two-way ANOVA (phase × exercise) followed by post hoc tests where appropriate. Data are presented as mean ± SEM (ZT3-sed *n* = 5; ZT3-exe *n* = 8; ZT15-sed *n* = 8; ZT15-exe *n* = 8). # Phase main effect; $ exercise main effect. Significance levels: #/$ < 0.05; ### *p* < 0.001.

To assess the impact of exercise timing and circadian phase on hepatic steatosis and systemic metabolic health, we analyzed hepatic lipid accumulation, hepatic TG content, and plasma biochemical parameters. Statistical analysis was performed using two-way ANOVA (phase × exercise). Oil Red O staining of liver sections revealed a clear reduction in lipid droplet size in exercise-trained groups compared to sedentary controls (Figure 8a). Quantitative analysis confirmed a significant main effect of exercise on the percentage of lipid droplet area (F(1, 19) = 4.78, *p* = 0.0416, partial η^2^ = 0.20, mean difference = 4.77, 95% CI [0.20, 9.33]; Figure 8b). A significant main effect of phase was also detected for hepatic TG content (F(1, 25) = 15.49, *p* = 0.0006, partial η^2^ = 0.38, mean difference = −162.70, 95% CI [−259.00, −66.30]), with lower TG levels observed in the ZT3 groups compared with the ZT15 groups (Figure 8c). Analysis of plasma liver function markers revealed a significant main effect of phase on AST (*p* = 0.0295; Figure 8d, Table S3), while no significant differences were observed for ALT or albumin (Figure 8e,f). Additional plasma biochemical analyses of lipid metabolism markers (T-Cho, LDL-C, HDL-C), myocardial/tissue damage markers (CK, LDH), pancreatic function markers (amylase, lipase), and renal function markers (BUN, Cr, UA) revealed no significant differences among groups (Figure S2).

Overall, these findings indicate that exercise was associated with reduced hepatic lipid accumulation, whereas circadian phase corresponded to variations in hepatic TG content and plasma AST levels.

### 3.4. Expression of Clock and Metabolic Genes in the Liver

To comprehensively characterize the phase- and exercise-dependent regulation of hepatic gene expression, we analyzed a panel of genes involved in circadian rhythms, lipid metabolism, and inflammation. Gene expression was analyzed by two-way ANOVA (phase × exercise), and multiple testing was controlled using the Benjamini–Hochberg false discovery rate (FDR) correction. Statistical significance was defined at q < 0.05. Full *p* and q values for all genes are provided in Supplementary Table S5.

#### 3.4.1. Core Clock Regulation (Figure 9a)

Among the core clock genes, *Bmal1* expression showed a significant main effect of phase (F(1, 25) = 36.26, *p* < 0.0001, partial η^2^ = 0.59, q = 0.0001), being markedly higher at ZT15 than at ZT3. Per2 expression also exhibited a significant phase effect (F(1, 25) = 6.76, *p* = 0.0155, partial η^2^ = 0.21, q = 0.0418), with higher expression at ZT3 than at ZT15, indicating elevated transcriptional activity during the rest phase. A trend toward an exercise effect (F(1, 25) = 4.21, *p* = 0.0507, partial η^2^ = 0.14) was observed, but this difference was not supported after FDR adjustment, with slightly higher expression in sedentary than in exercised mice. Likewise, *Cry1* expression demonstrated a robust phase effect (F(1, 25) = 19.63, *p* = 0.0002, partial η^2^ = 0.44, q = 0.0010), being significantly higher at ZT15 than at ZT3. *Nr1d1* also exhibited a significant main effect of phase (F(1, 25) = 19.80, *p* = 0.0002, partial η^2^ = 0.44, q = 0.0010), with higher expression in ZT3 than in ZT15. Although trends were detected for exercise (F(1, 25) = 3.97, *p* = 0.0573, partial η^2^ = 0.14) and for the phase × exercise interaction (F(1, 25) = 3.43, *p* = 0.0757, partial η^2^ = 0.12), neither reached significance after FDR correction. Post hoc analysis indicated that *Nr1d1* expression was significantly higher in ZT3-sed mice than in both ZT15-sed (mean difference = 0.90, t(25) = 4.19, 95% CI [0.46, 1.34], *p* = 0.0003, Hedges’ g = 1.63) and ZT3-exe groups (mean difference = 0.55, t (25) = 2.56, 95% CI [0.11, 0.99], *p* = 0.0170, Hedges’ g = 0.99).

**Figure 9:**
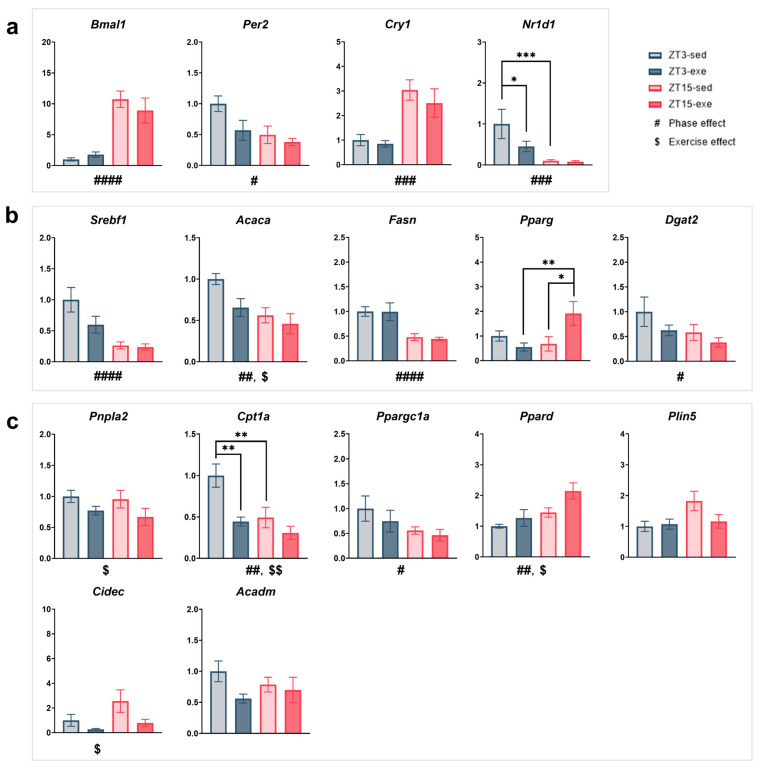
Effects of exercise timing and intervention on the expression of circadian and metabolism-related genes in mouse liver. (**a**) Core clock genes: *Bmal1*, *Per2*, *Cry1*, *Nr1d1*. (**b**) Lipid synthesis and transcriptional control: *Srebf1*, *Acaca*, *Fasn*, *Pparg*, *Dgat2*. (**c**) Lipid mobilization and oxidation: *Pnpla2*, *Cpt1a*, *Ppargc1a*, *Ppard*, *Plin5*, *Cidec*, *Acadm*. Statistical analysis was performed using two-way ANOVA (phase × exercise) followed by post hoc tests where appropriate. Data are presented as mean ± SEM (ZT3-sed *n* = 5; ZT3-exe *n* = 8; ZT15-sed *n* = 8; ZT15-exe *n* = 8). # Phase main effect; $ exercise main effect; * post hoc comparison. Significance levels: #/$/* *p* < 0.05; ##/$$/** *p* < 0.01; ###/*** *p* < 0.001; #### *p* < 0.0001.

Taken together, these results confirm that hepatic core clock gene expression retained robust circadian phase dependency under HFD conditions (all q < 0.05 for *Bmal1*, *Per2*, *Cry1*, and *Nr1d1*), whereas the effects of exercise and phase × exercise interaction were not significant after correction, suggesting that long-term exercise exerts only limited influence on hepatic clock regulation.

#### 3.4.2. Lipid Synthesis and Transcriptional Control (Figure 9b)

For lipid metabolism-related transcription factors, *Srebf1* showed a highly significant phase effect (F(1, 25) = 24.42, *p* < 0.0001, partial η^2^ = 0.49, q = 0.0001), with higher expression at ZT3 than at ZT15, and a trend toward an exercise effect (F(1, 25) = 3.72, *p* = 0.0653, partial η^2^ = 0.13) that was not confirmed after FDR adjustment. *Acaca* exhibited significant main effects of both phase and exercise before adjustment (phase: F(1, 25) = 8.59, *p* = 0.0071, partial η^2^ = 0.26; exercise: F(1, 25) = 4.27, *p* = 0.0494, partial η^2^ = 0.15). Only the phase effect remained significant after correction (q = 0.0246), with higher expression at ZT3 than at ZT15, whereas expression tended to be higher in sedentary than in exercised mice. *Fasn* expression showed a robust phase effect (F(1, 25) = 21.43, *p* < 0.0001, partial η^2^ = 0.46, q = 0.0001), with markedly higher levels at ZT3 than at ZT15. *Pparg* displayed a significant phase × exercise interaction before correction (F(1, 25) = 6.05, *p* = 0.0212, partial η^2^ = 0.19), but this interaction did not remain significant after FDR adjustment. Post hoc analysis nevertheless revealed higher expression in ZT15-exe mice than in both ZT3-exe (mean difference = −1.36, t(25) = 3.02, 95% CI [−2.29, −0.43], *p* = 0.0057, Hedges’ g = 1.17) and ZT15-sed groups (mean difference = −1.23, t(25) = 2.74, 95% CI [−2.16, −0.31], *p* = 0.0112, Hedges’ g = 1.06). Because the phase × exercise interaction did not remain significant after FDR correction, the pairwise post hoc results should be interpreted as exploratorily. Among TG synthesis-related genes, *Dgat2* exhibited a significant phase effect (F(1, 25) = 4.41, *p* = 0.0461, partial η^2^ = 0.15) and a trend toward an exercise effect (F(1, 25) = 3.34, *p* = 0.0795, partial η^2^ = 0.12), with higher expression at ZT3 and in sedentary mice; however, neither difference remained significant after FDR adjustment.

Overall, these results indicate that hepatic lipogenic and transcriptional regulatory genes are primarily governed by circadian phase (notably *Srebf1*, *Acaca*, and *Fasn*; all q < 0.05), whereas exercise-related and interaction effects were largely diminished after multiple-comparison correction, implying that long-term exercise only modestly influences hepatic lipid synthesis pathways.

#### 3.4.3. Lipid Mobilization and Oxidation (Figure 9c)

Regarding lipolysis-related genes, *Pnpla2* showed a significant main effect of exercise (F(1, 25) = 4.34, *p* = 0.0477, partial η^2^ = 0.15), with higher expression in sedentary than in exercised mice, although this difference was not supported after FDR adjustment. For fatty acid oxidation-related genes, *Cpt1a* exhibited clear main effects of both exercise (F(1, 25) = 13.42, *p* = 0.0012, partial η^2^ = 0.35, q = 0.0353) and phase (F(1, 25) = 10.11, *p* = 0.0039, partial η^2^ = 0.29, q = 0.0158), together with a trend toward an interaction (F(1, 25) = 3.37, *p* = 0.0783, partial η^2^ = 0.12), which did not remain significant after correction. Expression levels were higher at ZT3 and under sedentary conditions. Post hoc analysis confirmed that *Cpt1a* expression was significantly higher in ZT3-sed mice than in both ZT15-sed (mean difference = 0.51, t(25) = 3.34, 95% CI [0.19, 0.82], *p* = 0.005, Hedges’ g = 1.30) and ZT3-exe groups (mean difference = 0.56, t(25) = 3.65, 95% CI [0.24, 0.87], *p* = 0.0013, Hedges’ g = 1.42). *Ppard* showed significant main effects of phase (F(1, 25) = 8.17, *p* = 0.0085, partial η^2^ = 0.25) and exercise (F(1, 25) = 4.38, *p* = 0.0468, partial η^2^ = 0.15), being more highly expressed at ZT15 and in exercised groups. After FDR correction, only the phase effect remained significant (q = 0.0258), whereas the exercise effect was attenuated.

A modest exercise-related increase was also observed for *Acadm* (F(1, 25) = 2.99, *p* = 0.0960, partial η^2^ = 0.11), but this tendency was not statistically supported after adjustment. Among other oxidative and lipid droplet-associated genes, *Ppargc1a* showed higher expression at ZT3, whereas *Plin5* and *Cidec* tended to be elevated at ZT15. *Cidec* expression also appeared lower in exercised mice (Table S3). However, none of these minor differences reached statistical significance following FDR correction. In contrast, *Acox1*, *Plin2*, and *Mgll* showed no notable variation across groups (Figure S3b).

Overall, these findings highlight *Cpt1a* as the only oxidative gene retaining significant phase- and exercise-dependent regulation after multiple testing correction, whereas other genes displayed directionally consistent but statistically attenuated changes. This suggests that hepatic oxidative capacity is predominantly enhanced during the rest phase, with chronic exercise exerting only a modest additional influence on lipid mobilization.

#### 3.4.4. Other Metabolic Processes (Supplementary Figure S3c,d)

No significant differences were detected in genes associated with other metabolic processes. For fatty-acid transport and glycerolipid synthesis genes, including *Cd36*, *Slc27a2*, *Slc27a5*, and *Gpam*, expression levels were comparable across all groups. Similarly, genes involved in hepatic glucose metabolism and lipoprotein assembly, such as *Slc2a2*, *G6pc1*, *Apob*, and *Mttp* showed no phase- or exercise-dependent changes. Overall, genes involved in hepatic substrate transport and lipoprotein formation remained unaffected by either circadian phase or exercise, suggesting that these basal metabolic pathways are stably maintained under HFD conditions.

### 3.5. Expression of Clock and Metabolic Genes in EPI

To elucidate the molecular adaptations in EPI in response to exercise timing and circadian phase, we analyzed the expression of genes involved in lipogenesis, lipolysis, fatty acid oxidation, and adipokine signaling. Differences in gene expression were evaluated using two-way ANOVA (phase × exercise). To account for multiple testing across genes, *p* values were further adjusted using the Benjamini–Hochberg false discovery rate (FDR) method, with significance defined as q < 0.05. The complete set of FDR-adjusted results is presented in Supplementary Table S5.

#### 3.5.1. Core Clock Regulation (Figure 10a)

In EPI, the core clock genes *Bmal1* and *Cry1* displayed clear phase-dependent oscillations (*Bmal1*: F(1, 25) = 25.27, *p* < 0.0001, partial η^2^ = 0.50, q = 0.0001; *Cry1*: F(1, 25) = 39.58, *p* < 0.0001, partial η^2^ = 0.61, q = 0.0001), with markedly higher expression at ZT15 than at ZT3. Similarly, *Nr1d1* showed a significant main effect of phase (F(1, 24) = 21.53, *p* = 0.0001, partial η^2^ = 0.47, q = 0.0004), being more highly expressed at ZT15. In contrast, *Per2* showed only a weak trend (F(1, 25) = 2.98, *p* = 0.0967, partial η^2^ = 0.11), which was not supported after FDR correction. Collectively, these findings indicate that EPI core clock genes maintain robust circadian phase dependency under long-term HFD and exercise conditions (q < 0.05 for *Bmal1*, *Cry1*, and *Nr1d1*), while exercise effects were not statistically evident.

**Figure 10:**
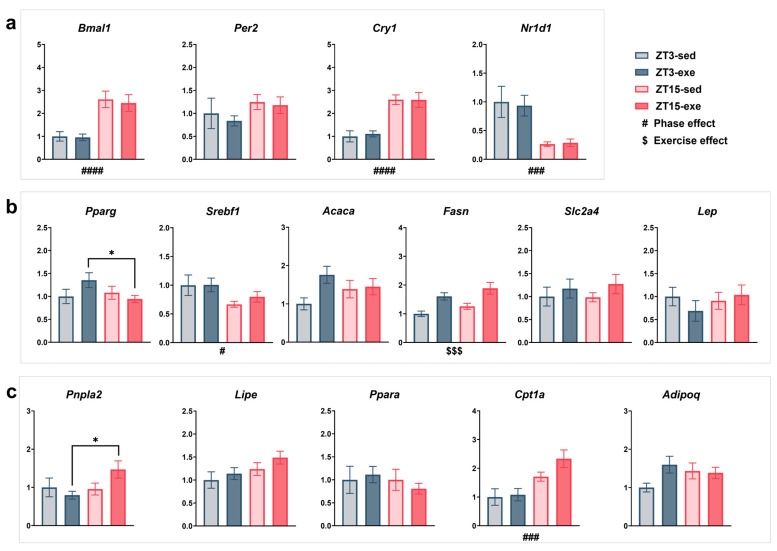
Effects of exercise timing on the expression of metabolism-related genes in EPI. (**a**) Core clock genes: *Bmal1*, *Per2*, *Cry1*, *Nr1d1*. (**b**) Lipogenesis and energy metabolism-related genes: *Pparg*, *Srebf1*, *Acaca*, *Fasn*, *Slc2a4*, *Lep*. (**c**) Lipolysis and fatty acid oxidation-related genes: *Pnpla2*, *Lipe*, *Cpt1a*, *Ppara*, *Adipoq*. Statistical analysis was performed using two-way ANOVA (phase × exercise). Data are presented as mean ± SEM (ZT3-sed *n* = 5; ZT3-exe *n* = 8; ZT15-sed *n* = 8; ZT15-exe *n* = 8). # Phase main effect; $ exercise main effect; * post hoc comparison. Significance levels: #/* *p* < 0.05; ###/$$$ *p* < 0.001; #### *p* < 0.0001.

#### 3.5.2. Lipogenesis and Energy Metabolism (Figure 10b)

To explore phase- and exercise-dependent changes in lipid synthesis, we next analyzed key lipogenic genes. A trend toward a phase × exercise interaction was observed for *Pparg* (F(1, 25) = 3.05, *p* = 0.0931, partial η^2^ = 0.11), with post hoc analysis showing significantly higher expression in ZT3-exe than in ZT15-exe mice (mean difference = 0.41, t(25) = 2.22, 95% CI [0.03–0.79], *p* = 0.0359, Hedges’ g = 0.86); however, this effect was not retained after FDR correction. *Srebf1* expression exhibited a significant main effect of phase (F(1, 25) = 6.17, *p* = 0.0201, partial η^2^ = 0.20, q = 0.0464), with higher levels at ZT3 than ZT15. *Acaca* tended to be higher in exercised than in sedentary mice (*p* = 0.0787), though this difference was not significant after correction. In contrast, *Fasn* was robustly reduced by exercise (F(1, 25) = 16.43, *p* = 0.0004, partial η^2^ = 0.40, q = 0.0059) and showed a mild phase trend (F(1, 25) = 3.17, *p* = 0.0874, partial η^2^ = 0.11), with slightly higher expression at ZT15. *Slc2a4* and *Lep* showed no significant changes across groups.

Overall, these findings suggest that long-term exercise significantly suppresses *Fasn* expression in EPI, while *Srebf1* retains phase-dependent rhythmicity. Other lipogenic and regulatory genes showed modest or statistically attenuated responses after multiple testing correction.

#### 3.5.3. Lipolysis and Fatty Acid Oxidation (Figure 10c)

We then assessed genes involved in lipid breakdown and oxidation. *Pnpla2* showed a trend toward a phase × exercise interaction (F(1, 25) = 3.67, *p* = 0.0672, partial η^2^ = 0.13), with post hoc analysis indicating significantly lower expression in ZT3-exe than in ZT15-exe (mean difference = −0.67, t(25) = 2.78, 95% CI [−1.17, −0.17], *p* = 0.0105, Hedges’ g = 1.08), and lower levels in ZT15-sed than in ZT15-exe mice (mean difference = −0.51, t(25) = 2.03, 95% CI [−1.03, −0.01], *p* = 0.0521, Hedges’ g = 0.79); however, this interaction did not remain significant after FDR correction. *Lipe* also showed a weak phase trend (F(1, 25) = 4.00, *p* = 0.0565, partial η^2^ = 0.14) but was not significant after adjustment. In contrast, *Cpt1a* exhibited a robust phase effect (F(1, 25) = 15.38, *p* = 0.0006, partial η^2^ = 0.38, q = 0.0017), showing higher expression at ZT15 than at ZT3. *Ppara*, *Adipoq*, and *Slc2a4* were unaffected by either phase or exercise.

Together, these findings highlight *Cpt1a* as the only oxidative gene maintaining a significant phase-dependent pattern after correction, indicating that lipid oxidation in EPI is primarily governed by circadian phase rather than by chronic exercise.

### 3.6. Correlations Between Plasma TG and Gene Expression Levels (Figure 11)

To further examine the relationships between plasma TG concentrations and the expression of lipid metabolism-related genes in liver and adipose tissue, we performed Pearson correlation and linear regression analyses. In EPI, both *Pnpla2* (r = −0.49, R^2^ = 0.24, slope = −9.27, 95% CI [−15.86, −2.68], F(1, 27) = 8.33, *p* = 0.0076) and *Lipe* (r = −0.42, R^2^ = 0.18, slope = −10.39, 95% CI [−19.22, −1.57], F(1,27) = 5.84, *p* = 0.02) showed significant negative correlations with plasma TGs, with higher expression levels corresponding to lower plasma TG values. In the liver, *Pparg* also displayed a significant negative correlation with plasma TGs (r = −0.52, R^2^ = 0.27, slope = −5.06, 95% CI [−8.35, −1.77], F(1, 27) = 9.97, *p* = 0.004), suggesting that lower hepatic *Pparg* expression was associated with lower plasma TG levels. Together, these results indicate a tissue-specific association between plasma lipid levels and the expression of lipid metabolism-related genes.

## 4. Discussion

### 4.1. Main Findings

This study systematically investigated how circadian phase influences long-term exercise adaptations in HFD-induced obese mice. Plasma TG was defined as the primary endpoint, representing the most sensitive systemic marker of metabolic adaptation. Three main observations emerged: First, plasma TG showed a significant circadian phase × exercise interaction, being lowest after training during the active phase (ZT15). Second, hepatic TG content was primarily determined by circadian phase, being lower in ZT3 groups, while exercise mainly reduced hepatic lipid droplet area. Third, active-phase exercise enhanced adipose expression of lipolysis- and oxidation-related genes, whereas rest-phase training coincided with a hepatic oxidative advantage. Overall, these findings indicate that exercise effects are both time- and tissue-dependent: active-phase exercise promotes lipid mobilization, while rest-phase conditions facilitate hepatic lipid clearance. Notably, several gene-level signals became weaker after multiple-comparison correction; therefore, these results should be interpreted as exploratory trends rather than definitive conclusions.

### 4.2. Behavioral and Systemic Phenotypes

Building on these primary findings, the metabolic effects of long-term exercise interventions are both time-dependent and tissue-specific, showing consistent patterns at the behavioral and systemic levels. Plasma TG displayed a clear phase × exercise interaction, being lowest in the active-phase exercise group (ZT15-exe), suggesting that the time dependency observed in acute exercise is preserved at the steady-state endpoint after long-term training [20]. Hepatic TG content was mainly governed by phase, with lower levels in ZT3 than in ZT15, while exercise primarily reduced lipid droplet area. Although the pattern of hepatic TG changes observed in this study differs slightly from that reported previously, possibly due to differences in training time points, both studies consistently suggest a phase-dependent influence of exercise timing on long-term metabolic adaptation [25]. In addition, while exercise timing affected body weight and feeding rhythms, these effects were insufficient to explain the observed metabolic outcomes. In the ZT15-exe group, the reduced amplitude of feeding rhythm may indicate either altered energy allocation or a partial attenuation of rhythmicity; however, further studies are required to clarify this relationship.

### 4.3. Hepatic Mechanisms

The results demonstrate that hepatic metabolic state is primarily governed by circadian phase and secondarily modulated by exercise. Hepatic TG content was significantly higher at ZT15 than at ZT3, indicating that lipid accumulation tends to occur during the active phase. Histologically, Oil Red O staining showed that hepatic lipid droplet area was markedly reduced in exercised mice, suggesting that long-term training facilitates hepatic lipid clearance and turnover. Meanwhile, *Cpt1a* expression was consistently higher in ZT3 than in ZT15 groups and in sedentary than in exercised mice, indicating strong circadian rhythmicity in hepatic fatty acid oxidation, with exercise potentially enhancing lipid breakdown on this background. Core clock genes and lipogenic regulators (*Bmal1*, *Cry1*, *Per2*, *Nr1d1*, *Srebf1*, *Acaca*, and *Fasn*) displayed clear phase-dependent expression, further confirming that hepatic lipogenesis networks are rhythmically regulated by intrinsic clock systems. This is consistent with previous circadian transcriptome atlases, which revealed time-of-day-dependent expression of lipid metabolism genes in the liver, highlighting that the endogenous clock coordinates lipid synthesis and oxidation to maintain metabolic homeostasis [34]. Moreover, *Pparg* expression was negatively correlated with plasma TGs, suggesting a potential transcriptional mechanism whereby exercise and circadian phase jointly influence hepatic lipid droplet homeostasis, although this mechanism requires further validation.

Overall, hepatic changes were mainly phase-driven, with exercise acting to optimize lipid handling and relieve metabolic stress rather than directly reducing total hepatic TGs. This finding is generally consistent with previous reports showing that late active-phase exercise more effectively attenuates hepatic steatosis than early active-phase exercise [25], although the specific response patterns observed in this study were not entirely identical.

### 4.4. Adipose Tissue Mechanisms

The results indicate that adipose tissue metabolism exhibits robust circadian characteristics and is further modulated by exercise timing. In EPI, core clock genes and *Srebf1* showed significant phase-dependent expression, reflecting rhythmic regulation of lipogenic pathways, while *Fasn* was elevated in exercised mice, suggesting that long-term training might contribute to increased de novo lipogenesis in adipose tissue. Such an adaptive remodeling likely reflects the tissue’s metabolic buffering role in systemic lipid homeostasis, facilitating lipid uptake and re-esterification to stabilize plasma TG levels [35,36]. This interpretation is consistent with the notion that healthy adipose tissue supports systemic metabolic health through dynamic lipid turnover [35]. *Pnpla2* expression was inversely associated with plasma TGs, implying that mild enhancement of adipose lipolytic activity may contribute to systemic lipid improvement.

In summary, long-term exercise may induce rhythmic adaptation in adipose tissue, rebalancing between “mobilization–oxidation” and “synthesis–storage” phases, thereby improving lipid handling and transport efficiency. This trend is consistent with Pendergrast et al. [20], who reported that active-phase exercise enhances fat mobilization and mitochondrial function, further supporting the tissue specificity of exercise timing effects.

### 4.5. Summary Model

Integrating these findings, we propose a “tissue × time” cooperative model: the metabolic benefits of exercise are jointly determined by tissue type and circadian phase, with the significant phase × exercise interaction in plasma TG concentration reflecting this systemic coordination. During the rest phase (ZT3), the liver exhibits stronger fatty acid oxidation, and exercise may act synergistically with this oxidative background to reduce lipid droplet load and optimize lipid handling. In contrast, during the active phase (ZT15), adipose tissue appears more responsive at the transcriptional level. Exercise increased *Fasn* expression, while *Pnpla2* and *Lipe* were inversely associated with plasma TG levels, suggesting rhythmic regulation of lipid uptake and re-esterification, which may underlie the observed TG reduction. Taken together, TG reduction following active-phase exercise may correspond to adipose transcriptional remodeling, whereas rest-phase exercise appears to reflect improved hepatic lipid processing.

### 4.6. Translational Implications

Our study provides preclinical evidence for the concept of “chrono-exercise,” indicating that the metabolic outcomes of exercise depend on the timing of training. Human studies likewise support this circadian dependence. Morning exercise enhances parasympathetic activity, whereas evening sessions increase sympathetic tone [37], sustain higher lipid utilization during recovery [38], and improve insulin sensitivity and lipid profiles more effectively than morning exercise [39]. In addition, exercise has been shown to modulate the circadian expression of core clock genes such as *Cry1*, *Per2*, and *Bmal1* in human skeletal muscle [40]. Collectively, these findings suggest that the optimal exercise time may vary depending on individual circadian characteristics and metabolic goals.

At the clinical level, circulating adipokines such as adiponectin and leptin are measurable indicators of metabolic health. Although this study examined adipokines only at the transcriptional level and found no significant group differences, clinical evidence indicates that decreased leptin and increased resistin levels are associated with malnutrition and dyslipidemia [41]. These observations emphasize the potential value of circadian-aligned exercise programs in preventing metabolic disorders. Overall, the proposed “tissue × time” model provides a framework for integrating circadian rhythms with exercise physiology, but further mechanistic and clinical validation across sexes and populations is required to establish its broader applicability.

### 4.7. Limitations

Several limitations should be acknowledged. First, sampling was performed 48 h after the final exercise session following a 4 h fast, which minimized acute effects but limited the ability to infer dynamic lipid flux. Therefore, the mechanistic interpretations should be considered hypothesis-generating and require further validation. Second, protein-level or functional analyses (e.g., ATGL, HSL, CPT1A) were not conducted, reducing the certainty of pathway-related conclusions. Third, circadian phase was mainly inferred from feeding behavior, while other physiological rhythms were not monitored, limiting system-level interpretation. Fourth, the light–dark manipulation used for ZT15 mice may have introduced phase-specific confounding despite re-entrainment before the experiment. Fifth, forced treadmill exercise may have induced stress responses interacting with circadian regulation. Finally, this study was limited to male C57BL/6J mice with modest sample sizes, and the findings should therefore be regarded as preclinical and hypothesis-generating, warranting further confirmation across sexes, strains, and human populations.

## 5. Conclusions

In conclusion, exercise timing influenced the long-term metabolic adaptations in obese mice. Plasma TGs were lowest after active-phase training (ZT15), suggesting a potential advantage in systemic lipid regulation, whereas rest-phase exercise (ZT3) was accompanied by higher hepatic oxidative activity. These findings indicate tissue-specific metabolic responses across circadian phases, supporting a descriptive “tissue × time” framework. Given the experimental conditions, mechanistic interpretations remain to be validated, and future studies on both sexes and human populations are needed to clarify circadian-dependent exercise effects.

## Figures and Tables

**Figure 11:**
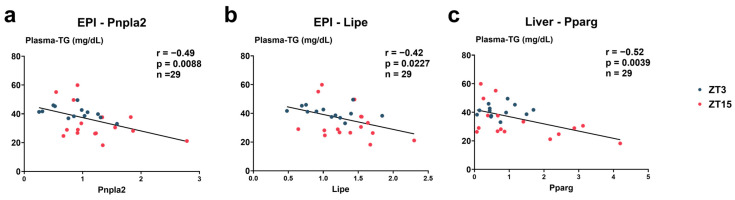
Correlations between plasma TG and gene expression levels in EPI and liver. (**a**–**c**) Scatter plots showing linear regression analyses between plasma TG concentrations and the expression levels of *Pnpla2* (**a**), *Lipe* (**b**), and *Pparg* (**c**). Data are presented as individual values from ZT3 (*n* = 13) and ZT15 (*n* = 16). Statistical analysis was performed using linear regression (Pearson correlation, least-squares method). Solid lines indicate the best-fit regression. The correlation coefficient (*r*) and *p* value are shown in each panel. Negative slopes indicate that higher expression of *Pnpla2*, *Lipe*, and *Pparg* was associated with lower plasma TG concentrations.

**Table 1:** Primer sequence for real-time PCR analysis.

Gene	Forward (5′-3′)	Reverse (3′-5′)
*Acaca*	GGCTCGTGTGTGGAAGTGGATG	GTGGTGTAACTGCTGCCGTCAT
*Acadm*	TGTTAATCGGTGAAGGAGCAG	CTATCCAGGGCATACTTCGTG
*Acox1*	GGGGAACATCATCACAGGGG	ATCATAGCGGCCGAGAACAG
*Adipoq*	GCCGCTTATGTGTATCGCTCAG	CTTGCCAGTGCTGCCGTCATA
*Apob*	GCCTTCCAGTTGGCAACACAGT	GGCAGGTCAACATCGGCAATCA
*Bmal1*	GGACTTCGCCTCTACCTGTT	CCTCGTTGTCTGGCTCATTG
*Cd36*	TGCGACATGATTAATGGCACAGAC	TCCGAACACAGCGTAGATAGACCT
*Cidec*	TGTCGTGTTAGCACCGCAGAT	GCCATCTTCCTCCAGCACCA
*Cpt1a*	CAAGCCAGACGAAGAACA	TGACCATAGCCATCCAGAT
*Cry1*	GCTGGCGTGGAAGTCATCGT	ATGGTGTCTGCTGGCATCTCC
*Dgat2*	GCACCCGACCCAGAAAGACATC	AGTTCACCTCCAGCACCTCAGT
*Fasn*	ACTCAAGTGGCTGATGTG	TGCTGTCGTCTGTAGTCT
*G6pc1*	GTCGTGGCTGGAGTCTTGT	CGGAGGCTGGCATTGTAGA
*Gapdh*	TCTCCTGCGACTTCAACA	TGTAGCCGTATTCATTGTCA
*Gpam*	TGAGCAGCAGCAGAGTCCAAGA	GTTCAACTCCGCAGCCACTTCA
*Lep*	TTCACACACGCAGTCGGTATCC	AGGCTGGTGAGGACCTGTTGAT
*Lipe*	CTGAGATTGAGGTGCTGTC	GGTGAGATGGTAACTGTGAG
*Mgll*	TGATTTCACCTCTGGTCCTTG	GTCAACCTCCGACTTGTTCC
*Mlxipl*	GCAACCACGCTTCAGAAGACAG	GCTGCTGGCACAAGTTGATGG
*Mttp*	CAGCGTCCACATACAGCCTTGA	TCCTCAGAATGCCAGAGCCAGA
*Nr1d1*	ACGGCAAGGCAACACCAA	GCGGCTCAGGAACATCACT
*Per2*	GCTGCGGATGCTCGTGGAAT	GGTTGTGCTCTGCCTCTGTCAT
*Plin2*	GCAACAGAGCGTGGTGATGAGA	CGGAGGACACAAGGTCGTAGGT
*Plin5*	ACCGCTTCCTGCCCATGACT	TTGCTGCCTCTGCTCCTCCA
*Pnpla2*	TTCAGACAACTTGCCACTT	CGGTAGAGATTGCGAAGG
*Ppara*	ACGATGCTGTCCTCCTTGAT	AACGGCTTCCTCAGGTTCTTA
*Ppard*	GCTGCTGCAGAAGATGGCA	CACTGCATCATCTGGGCATG
*Pparg*	CGTGAAGCCCATCGAGGACATC	TGGAGCACCTTGGCGAACAG
*Ppargc1a*	GTCGTGGCTGGAGTCTTGT	CGGAGGCTGGCATTGTAGA
*Rps18*	TTCTGGCCAACGGTCTAGACAAC	CCAGTGGTCTTGGTGTGCTGA
*Slc27a2*	ACCACAGAAGTCGCTGACATCG	GCACAGGCACGCCATACACAT
*Slc27a5*	CCTTGTGCTGCTTGGCTTGG	GGTGGCTGTAGAGGCAATAGGA
*Slc2a2*	TTGACTGGAGCCCTCTTGATGG	CTGAGTGTGGTTGGAGCGATCT
*Slc2a4*	GCTGGTGTGGTCAATACGGTCT	GCAGAGCCACGGTCATCAAGAT
*Srebf1*	GCCATCGACTACATCCGCTTCT	TGCCTCCTCCACTGCCACAA

Forward (5′ → 3′) and reverse (3′ → 5′) primer sequences used for quantitative real-time PCR (qPCR) analysis are listed. The target genes cover several functional categories: Housekeeping genes (*Gapdh*, *Rps18*). Circadian clock genes (*Bmal1*, *Cry1*, *Per2*, *Nr1d1*). Lipid metabolism-related genes (*Acaca*, *Acadm*, *Acox1*, *Apob*, *Cd36*, *Cidec*, *Cpt1a*, *Dgat2*, *Fasn*, *Gpam*, *Lipe*, *Mgll*, *Mttp*, *Plin2*, *Plin5*, *Pnpla2*, *Slc27a2*, *Slc27a5*, *Slc2a2*, *Slc2a4*, *Srebf1*). Transcriptional regulators and nuclear receptors (*Ppara*, *Ppard*, *Pparg*, *Ppargc1a*, *Mlxipl*). Adipokines and hormones (*Adipoq*, *Lep*). Gene expression analyses were performed using RNA samples from 5 to 8 mice per group. Full gene names are provided in the Abbreviations section.

## Data Availability

The original contributions presented in this study are included in the article and Supplementary Materials. Further inquiries can be directed to the corresponding author.

## Supplementary Materials

The following supporting information can be downloaded at: https://www.mdpi.com/article/10.3390/nu17203281/s1, Table S1: Feeding ratio (Active/Rest) during high-fat diet feeding; Table S2: Feeding ratio (Active/Rest) during exercise; Table S3: Summary of statistical analyses for tissue weights, plasma parameters, and gene expression; Table S4: ANCOVA results using Week 2 (Day 12) and Week 8 body weight as covariates (plasma parameters); Table S5: Summary of two-way ANOVA and FDR-adjusted results for liver and epididymal adipose gene expression (EPI); Figure S1: Absolute tissue weights; Figure S2: Plasma biochemical parameters in mice; Figure S3: Effects of exercise timing and intervention on the expression of metabolism-related genes in mouse liver.

## Abbreviations

The following abbreviations are used in this manuscript:

A/R	Active/Rest ratio
*Acaca*	Acetyl-Coenzyme A carboxylase alpha
*Acadm*	Acyl-Coenzyme A dehydrogenase, medium chain
*Acox1*	Acyl-Coenzyme A oxidase 1, palmitoyl
*Adipoq*	Adiponectin, C1Q and collagen domain containing
ALT	Alanine aminotransferase
ANCOVA	Analysis of covariance
ANOVA	Analysis of variance
*Apob*	Apolipoprotein B
AST	Aspartate aminotransferase
BAT	Brown adipose tissue
*Bmal1*	Basic helix-loop-helix ARNT like 1
BUN	Blood urea nitrogen
*Cd36*	CD36 molecule
cDNA	Complementary deoxyribonucleic acid
CI	Confidence interval
*Cidec*	Cell death-inducing DFFA-like effector c
CK	Creatine kinase
*Cpt1a*	Carnitine palmitoyltransferase 1a, liver
Cr	Creatinine
*Cry1*	Cryptochrome circadian regulator 1
*Dgat2*	Diacylglycerol O-acyltransferase 2
EPI	Epididymal white adipose tissue
*Fasn*	Fatty acid synthase
FDR	False discovery rate
*G6pc1*	Glucose-6-phosphatase catalytic subunit 1
gDNA	Genomic deoxyribonucleic Acid
*Gapdh*	Glyceraldehyde-3-phosphate dehydrogenase
GAS	Gastrocnemius
*Gpam*	Glycerol-3-phosphate acyltransferase, mitochondrial
HDL-C	High-density lipoprotein cholesterol
HFD	High-fat diet
LDH	Lactate dehydrogenase
LDL-C	Low-density lipoprotein cholesterol
*Lep*	Leptin
*Lipe*	Lipase, hormone sensitive
MASLD	Metabolic dysfunction-associated steatotic liver disease
*Mgll*	Monoglyceride lipase
*Mlxipl*	MLX interacting protein-like
*Mttp*	MLX interacting protein-like
NAFLD	Non-alcoholic fatty liver disease
NEFAs	Non-esterified fatty acids
*Nr1d1*	Nuclear receptor subfamily 1, group D, member 1
PBS	Phosphate-buffered saline
*Per2*	Period circadian clock 2
*Plin2*	Perilipin 2
*Plin5*	Perilipin 5
*Pnpla2*	Patatin-like phospholipase domain containing 2
*Ppara*	Peroxisome proliferator activator receptor alpha
*Ppard*	Peroxisome proliferator activator receptor delta
*Pparg*	Peroxisome proliferator activated receptor gamma
*Ppargc1a*	Peroxisome proliferative activated receptor, gamma, coactivator 1 alpha
qPCR	Quantitative real-time PCR
QUA	Quadriceps
RET	Retroperitoneal white adipose tissue
RNA	Ribonucleic acid
*Rps18*	Ribosomal protein S18
SEM	Standard error of the mean
*Slc27a2*	Solute carrier family 27 (fatty acid transporter), member 2
*Slc27a5*	Solute carrier family 27 (fatty acid transporter), member 5
*Slc2a2*	Solute carrier family 2
*Slc2a4*	Solute carrier family 4
*Srebf1*	Sterol regulatory element binding transcription factor 1
T-Cho	Total cholesterol
TGs	Triglycerides
UA	Uric acid
VLDL	Very-low-density lipoprotein
VO_2_ max	Maximal oxygen uptake;
WAT	White adipose tissue
ZT	Zeitgeber time
