# Supplementary material for: Circadian Phase Determines Tissue-Specific Adaptations to Long-Term Exercise in Obese Mice
Source: Nutrients. 2025 Oct 18;17(20):3281. doi: 10.3390/nu17203281 (PMC12567273; doi:10.3390/nu17203281)
Supplement: Supplementary file 1 [file nutrients-17-03281-s001.zip › nutrients-3926721-supplementary.pdf]

Table S1. Feeding ratio (Active/Rest) during high-fat diet feeding

	ZT3			ZT15			Unpaired t test with Welch's correction				
**Day**	**Mean**	**SEM**	**N**	**Mean**	**SEM**	**N**	**t(df)**	**p value**	**Mean diff. (95% CI)**	**Hedges' g**	**Sig.**
0	2.17	0.38	6	0.44	0.07	6	t(5.33) = 6.41	0.0011	−1.73 [−2.41, −1.05]	1.37	**
1	3.20	0.85	6	0.34	0.05	6	t(5.04) = 4.76	0.0050	−2.86 [−4.40, −1.32]	1.03	**
2	3.26	0.63	6	0.44	0.03	6	t(5.02) = 6.33	0.0014	−2.81 [−3.96, −1.67]	1.37	**
6	3.14	0.41	6	1.15	0.13	6	t(5.94) = 6.49	0.0007	−1.98 [−2.73, −1.23]	1.33	***
7	3.10	0.23	6	1.36	0.24	6	t(9.98) = 7.36	<0.0001	−1.74 [−2.27, −1.21]	1.24	****
11	3.25	0.38	6	1.84	0.24	6	t(8.43) = 4.40	0.0020	−1.41 [−2.15, −0.68]	0.79	**
12	3.21	0.51	6	2.57	0.59	6	t(9.77) = 1.16	0.2742	−0.64 [−1.87, 0.59]	0.20	ns
16	3.36	0.41	6	2.8	0.24	6	t(8.09) = 1.67	0.1329	−0.56 [−1.34, 0.21]	0.31	ns
21	3.14	0.73	6	2.47	0.27	6	t(6.40) = 1.22	0.2662	−0.67 [−2.00, 0.66]	0.24	ns
27	2.24	0.24	6	2.36	0.18	6	t(9.28) = 0.55	0.5968	−0.12 [−0.59, 0.36]	0.10	ns
35	3.61	1.27	6	1.96	0.27	6	t(5.46) = 1.80	0.1270	−1.66 [−3.97, 0.65]	0.38	ns
42	2.58	0.31	6	2.28	0.24	6	t(9.34) = 1.07	0.3138	−0.29 [−0.33, 0.91]	0.19	ns

Feeding data were analyzed as cage-level averages (n = 6 cages per group). Statistical comparisons between ZT3 and ZT15 groups were conducted using Welch's t-test for each measurement day. Values are expressed as mean ± SEM. Effect sizes (Hedges' g) and 95% confidence intervals (CIs) of the mean difference are reported to indicate the magnitude and precision of effects. Significance levels: ** p < 0.01; *** p < 0.001; **** p < 0.0001; ns, not significant. Positive mean differences indicate ZT3 > ZT15. Day 1 represents the day of light–dark reversal. ZT, Zeitgeber time; SEM, Standard error of the mean.

Table S2. Feeding ratio (Active/Rest) during exercise

		ZT3			ZT15			Two-way ANOVE					
**Day**		**Mean**	**SEM**	**N**	**Mean**	**SEM**	**N**	**Main effect**	**F(df)**	**p value**	**Mean diff. (95% CI)**	**partial η^2**	**Sig.**
55	sed	3.12	0.7	3	2.49	0.37	3	Exercise	F(1,8)=2.42	0.1586	0.67 [−0.32, 1.66]	0.23	ns
	exe	2.49	0.27	3	1.78	0.19	3	Phase	F(1,8)=2.44	0.1567	0.67 [−0.32, 1.65]	0.23	ns
56	sed	2.96	0.5	3	2.07	0.21	3	Exercise	F(1,8)=5.32	0.0500	0.61 [−0.10, 1.31]	0.40	*
	exe	2.16	0.26	3	1.65	0.10	3	Phase	F(1,8)=3.96	0.0818	0.70 [0.00, 1.41]	0.33	ns
57	sed	3.48	0.96	3	1.9	0.33	3	Exercise	F(1,8)=1.32	0.2836	0.65 [−0.53, 1.84]	0.14	ns
	exe	1.83	0.11	3	2.23	0.14	3	Phase	F(1,8)=1.62	0.2390	0.59 [−0.60, 1.78]	0.17	ns
62	sed	4.05	0.89	3	1.74	0.36	3	Exercise	F(1,8)=14.43	0.0052	0.62 [−0.55, 1.78]	0.64	**
	exe	3.04	0.31	3	1.51	0.07	3	Phase	F(1,8)=1.48	0.2579	1.92 [0.75, 3.08]	0.16	ns
63	sed	3.87	0.65	3	1.82	0.33	3	Exercise	F(1,8)=17.57	0.0030	0.66 [−0.28, 1.59]	0.69	**
	exe	2.86	0.34	3	1.51	0.12	3	Phase	F(1,8)=2.60	0.1453	1.70 [0.77, 2.64]	0.25	ns
70	sed	3.96	1.45	3	2.14	0.31	3	Exercise	F(1,8)=2.12	0.1839	1.02 [−0.78, 2.81]	0.21	ns
	exe	2.25	0.29	3	1.81	0.36	3	Phase	F(1,8)=1.71	0.2269	1.13 [−0.68, 2.93]	0.18	ns
77	sed	4.02	0.6	3	2.54	0.30	3	Exercise	F(1,8)=10.02	0.0133	1.32 [0.33, 2.30]	0.56	*
	exe	2.57	0.51	3	1.35	0.15	3	Phase	F(1,8)=9.53	0.0149	1.35 [0.37, 2.33]	0.54	*
84	sed	2.53	0.65	3	1.67	0.21	3	Exercise	F(1,8)=1.25	0.2962	0.44 [−0.39, 1.26]	0.14	ns
	exe	1.64	0.15	3	1.69	0.15	3	Phase	F(1,8)=1.46	0.2608	0.40 [−0.43, 1.23]	0.15	ns
91	sed	3.53	0.94	3	1.94	0.35	3	Exercise	F(1,8)=6.06	0.0392	0.68 [−0.64, 2.00]	0.43	*
	exe	2.66	0.54	3	1.44	0.14	3	Phase	F(1,8)=1.41	0.2687	1.41 [0.09, 2.73]	0.15	ns
98	sed	6.50	3.72	3	1.93	0.21	3	Exercise	F(1,8)=2.20	0.1759	2.19 [−2.24, 6.62]	0.22	ns
	exe	2.59	0.93	3	1.46	0.23	3	Phase	F(1,8)=1.30	0.2875	2.85 [−1.58, 7.28]	0.14	ns
105	sed	3.05	0.56	3	2.2	0.45	3	Exercise	F(1,8)=4.98	0.0563	0.51 [−0.35, 1.36]	0.38	ns
	exe	2.52	0.16	3	1.71	0.05	3	Phase	F(1,8)=1.87	0.2088	0.83 [−0.03, 1.68]	0.19	ns

Feeding data were analyzed as cage-level averages (n = 3 cages per group). Statistical analysis was performed using two-way ANOVA (factors: phase and exercise). Reported values represent main effects; interaction effects were tested but were not significant and are therefore omitted. Values are expressed as mean ± SEM. Partial η^2 values are presented as measures of effect size (0.01 ⩽ small, 0.06 ⩽ medium, and ⩾ 0.14 large effects).Effect sizes and 95% confidence intervals (CIs) of the mean differences are provided to indicate the magnitude and precision of the effects. Significance levels: * $p < 0.05$; ** $p < 0.01$; ns, not significant. ANOVA, Analysis of variance.

Table S3. Summary of statistical analyses for tissue weights, plasma parameters, and gene expression

Analyte	Effect / Comparison	F(df) or t(df)	Mean difference	p value	Effect size	95% CI	Sig.
RET (Rrelative)	Exercise effect	F(1,25)=3.20	4.00	0.0858	partial η²=0.11	[−0.61, 8.61]	trend
BAT (Rrelative)	Interaction	F(1,25)=3.43	1.12	0.0759	partial η²=0.12	[−0.13, 2.37]	trend
BAT (Rrelative)	ZT3-exe vs. ZT15-exe	t(25)=2.17	−0.87	0.0397	Hedges' g=0.84	[−1.69, −0.04]	*
Heart (Rrelative)	Phase effect	F(1,25)=4.93	0.76	0.0357	partial η²=0.16	[0.06, 1.47]	*
Heart (Absolute)	Interaction	F(1,25)=5.70	−48.04	0.0248	partial η²=0.19	[−89.47, −6.61]	*
Heart (Absolute)	ZT3-exe vs. ZT15-exe	t(25)=2.97	39.45	0.0064	Hedges' g=1.18	[12.13, 66.77]	**
Heart (Absolute)	ZT3-sed vs. ZT3-exe	t(25)=1.94	−29.34	0.0637	Hedges' g=0.78	[−60.49, 1.81]	trend
Kidney (Absolute)	Interaction	F(1,25)=3.14	−81.29	0.0886	partial η²=0.11	[−175.80, −13.19]	trend
Kidney (Absolute)	ZT3-exe vs. ZT15-exe	t(25)=1.93	58.24	0.0656	Hedges' g=0.77	[−4.06, 120.50]	trend
Kidney (Absolute)	ZT3-sed vs. ZT3-exe	t(25)=1.76	−60.74	0.0904	Hedges' g=0.70	[−131.80, 10.29]	trend
Glucose (W8 tail)	ZT3 vs. ZT15	t(27)=3.45	−24.50	0.0019	Hedges' g=1.34	[−39.60, −9.93]	**
AST (plasma)	Phase effect	F(1,25)=5.33	−34.88	0.0295	partial η²=0.18	[−65.98, −3.77]	*
Ppargc1a (liver)	Phase effect	F(1,25)=4.50	0.36	0.0441	partial η²=0.15	[0.01, 0.712]	*
Plin5 (liver)	Phase effect	F(1,25)=3.51	−0.46	0.0726	partial η²=0.12	[−0.96, 0.045]	trend
Cidec (liver)	Exercise effect	F(1,25)=4.84	1.26	0.0372	partial η²=0.16	[0.080, 2.43]	*
Cidec (liver)	Phase effect	F(1,25)=3.30	−1.04	0.0815	partial η²=0.12	[−2.21, 0.14]	trend
Acaca (EPI)	Exercise effect	F(1,25)=3.36	−0.41	0.0787	partial η²=0.12	[−0.87, 0.05]	trend

Statistical analyses were conducted using Welch's *t*-test for two-group comparisons (ZT3 n = 13, ZT15 n = 16) and two-way ANOVA (phase × exercise) for four-group comparisons (ZT3-sed n = 5, ZT3-exe n = 8, ZT15-sed n = 8, ZT15-exe n = 8), followed by post hoc tests where appropriate. Values are expressed as mean ± SEM. Reported statistics include *F*(df) and *t*(df) values, p values, effect sizes (partial η^2 or Hedges' *g*), and 95% confidence intervals (CIs), indicating the magnitude and precision of the observed effects. Significance levels: * $p < 0.05$; ** $p < 0.01$. Blood glucose was determined using capillary blood obtained from the tail tip. Rrelative, Rrelative tissue weights; Absolute, Absolute tissue weights; RET, Retroperitoneal white adipose tissue; BAT, Brown adipose tissue; W8, Week 8; AST, Aspartate aminotransferase. EPI, Epididymal white adipose tissue.

Table S4. ANCOVA results using Week 2 (Day 12) and Week 8 body weight as covariates (plasma parameters).

Dependent variable	Covariate	Source	F(df)	p value	Sig.
Plasma TG	Body weight W2	Group	F(3,24)=4.45	0.0127	*
		Covariate	F(1,24)=1.60	0.2185	ns
	Body weight W8	Group	F(3,24)=5.42	0.0054	**
		Covariate	F(1,24)=3.98	0.0576	ns
Plasma Glucose	Body weight W2	Group	F(3,24)=2.52	0.0823	ns
		Covariate	F(1,24)=0.96	0.3375	ns
	Body weight W8	Group	F(3,24)=2.73	0.0664	ns
		Covariate	F(1,24)=2.86	0.1037	ns
Plasma NEFA	Body weight W2	Group	F(3,24)=2.52	0.0821	ns
		Covariate	F(1,24)=0.03	0.8563	ns
	Body weight W8	Group	F(3,24)=1.74	0.1849	ns
		Covariate	F(1,24)=0.66	0.4237	ns

ANCOVA was performed for plasma parameters to evaluate group effects after adjusting for baseline body weight (ZT3-sed n = 5, ZT3-exe n = 8, ZT15-sed n = 8, ZT15-exe n = 8). Covariates included Week 2 body weight (W2, end of the adaptation period) and Week 8 body weight (W8, pre-exercise). Reported values represent adjusted *F*(df) and p values for both the main effect of group and the covariate (body weight). Significance levels: * $p < 0.05$; ** $p < 0.01$; ns, not significant. TG, Triglycerides; NEFA, Non-esterified fatty acids.

Table S5. Summary of two-way ANOVA and FDR-adjusted results for liver and epididymal adipose gene expression (EPI).

	Gene	Phase			Exercise			Interaction		
		p	q	Sig.	p	q	Sig.	p	q	Sig.
Liver	*Acaca*	0.0071	0.0246	*	0.0494	0.2400	ns	0.2735	0.9253	ns
	Acadm	0.8037	0.7215	ns	0.0960	0.2766	ns	0.2580	0.9253	ns
	Acox1	0.6991	0.6693	ns	0.1238	0.2899	ns	0.8527	1.0000	ns
	Apob	0.4375	0.5303	ns	0.2476	0.3587	ns	0.8324	1.0000	ns
	Bmal1	0.0000	0.0001	****	0.7067	0.7420	ns	0.3625	0.9353	ns
	Cd36	0.6200	0.6534	ns	0.2804	0.3587	ns	0.7254	1.0000	ns
	Cidec	0.0815	0.1520	ns	0.0372	0.2400	ns	0.3686	0.9353	ns
	Cpt1a	0.0039	0.0158	*	0.0012	0.0353	*	0.0783	0.7711	ns
	Cry1	0.0002	0.0010	***	0.4206	0.4705	ns	0.6551	1.0000	ns
	Dgat2	0.0461	0.1016	ns	0.0795	0.2597	ns	0.5823	1.0000	ns
	Fasn	0.0000	0.0001	****	0.8558	0.8676	ns	0.8929	1.0000	ns
	Glpc1	0.2689	0.3621	ns	0.1930	0.3546	ns	0.9585	1.0000	ns
	Gpam	0.1003	0.1703	ns	0.2335	0.3587	ns	0.8060	1.0000	ns
	Mgll	0.3386	0.4320	ns	0.2616	0.3587	ns	0.7154	1.0000	ns
	Mlxipl	0.7179	0.6693	ns	0.1035	0.2766	ns	0.8514	1.0000	ns
	Mttp	0.6760	0.6693	ns	0.2806	0.3587	ns	0.6838	1.0000	ns
	Nr1d1	0.0002	0.0010	***	0.0573	0.2400	ns	0.0757	0.7711	ns
	Per2	0.0155	0.0418	*	0.0507	0.2400	ns	0.2486	0.9253	ns
	Plin2	0.1493	0.2129	ns	0.1388	0.2899	ns	0.4290	1.0000	ns
	Plin5	0.0726	0.1467	ns	0.2394	0.3587	ns	0.1446	0.8806	ns
	Pnpla2	0.5636	0.6321	ns	0.0477	0.2400	ns	0.8210	1.0000	ns
	Ppara	0.9354	0.7819	ns	0.3257	0.3830	ns	0.4909	1.0000	ns
	Ppard	0.0085	0.0258	*	0.0468	0.2400	ns	0.3589	0.9353	ns
	Pparg	0.1390	0.2106	ns	0.2600	0.3587	ns	0.0212	0.6455	ns
	Ppargc1a	0.0441	0.1016	ns	0.3182	0.3830	ns	0.6387	1.0000	ns
	Slc27a2	0.5737	0.6321	ns	0.1321	0.2899	ns	0.9489	1.0000	ns
	Slc27a5	0.9126	0.7819	ns	0.4321	0.4705	ns	0.9403	1.0000	ns
	Slc2a2	0.1054	0.1703	ns	0.1479	0.2899	ns	0.2684	0.9253	ns
	Srebf1	0.0000	0.0001	****	0.0653	0.2400	ns	0.1013	0.7711	ns
	Bmal1	0.0000	0.0001	****	0.7467	0.8716	ns	0.8511	0.9890	ns
	Cry1	0.0000	0.0001	****	0.8444	0.8716	ns	0.8099	0.9890	ns
EPI	*Acc1*	0.8660	0.6668	ns	0.0787	0.5745	ns	0.1335	0.5257	ns
	Adipin	0.5753	0.5111	ns	0.1714	0.5745	ns	0.1080	0.5257	ns
	Cpt1a	0.0006	0.0017	**	0.1711	0.5745	ns	0.2875	0.8290	ns
	Fasn	0.0874	0.1241	ns	0.0004	0.0059	**	0.9419	0.9890	ns
	Lep	0.5581	0.5111	ns	0.6762	0.8716	ns	0.3158	0.8290	ns
	Lipe	0.0565	0.1088	ns	0.1960	0.5745	ns	0.7089	0.9890	ns
	Nr1d1	0.0001	0.0004	****	0.8894	0.8716	ns	0.7666	0.9890	ns
	Per2	0.0967	0.1241	ns	0.4541	0.7417	ns	0.9064	0.9890	ns
	Pnpla2	0.0825	0.1241	ns	0.4362	0.7417	ns	0.0672	0.5257	ns
	Ppara	0.4596	0.4826	ns	0.8469	0.8716	ns	0.4598	0.9890	ns
	Pparg	0.2495	0.2882	ns	0.4435	0.7417	ns	0.0931	0.5257	ns
	Slc2a4	0.8270	0.6668	ns	0.2345	0.5745	ns	0.7651	0.9890	ns
	Srebf	0.0201	0.0464	*	0.5345	0.7857	ns	0.5687	0.9890	ns

Two-way ANOVA (phase × exercise) was performed for each gene to assess the main and interaction effects of circadian phase and exercise training (ZT3-sed n = 5; ZT3-exe n = 8;

ZT15-sed n = 8; ZT15-exe n = 8). Reported values include raw p values and corresponding false discovery rate (FDR)–adjusted q values calculated by the Benjamini–Hochberg method. Significance summaries are indicated for phase, exercise, and interaction effects separately. Significance levels: * $q < 0.05$; ** $q < 0.01$; *** $q < 0.001$; **** $q < 0.0001$; ns, not significant. Abbreviations: sed, sedentary; exe, exercised; FDR, false discovery rate.

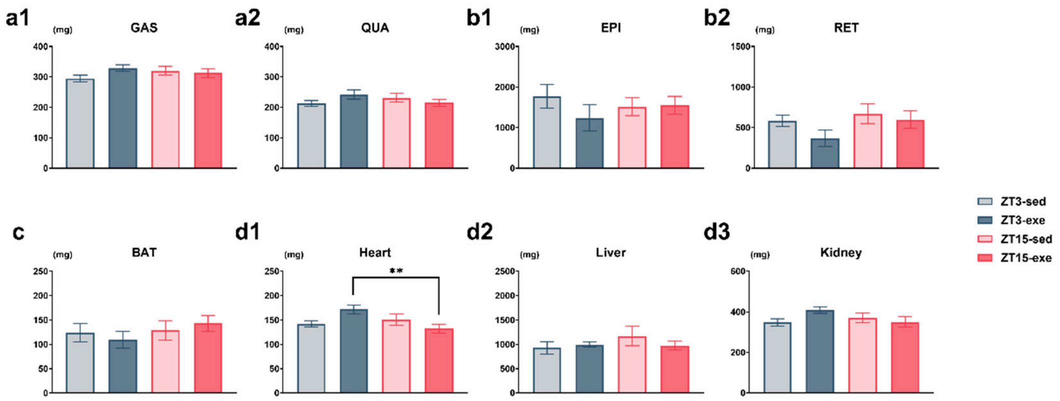

Figure S1. Absolute tissue weights.

(a) Skeletal muscle: gastrocnemius (a1), quadriceps (a2); (b) White adipose tissue (WAT): epididymal fat (b1), retroperitoneal fat (b2); (c) Brown adipose tissue (BAT); (d) Organs: heart (d1), liver (d2), kidney (d3). Data are presented as mean ± SEM (ZT3-sed n = 5; ZT3-exe n = 8; ZT15-sed n = 8; ZT15-exe n = 8). Statistical analysis was performed using two-way ANOVA (phase × exercise). * post hoc comparison. Significance levels: ** $p < 0.01$.

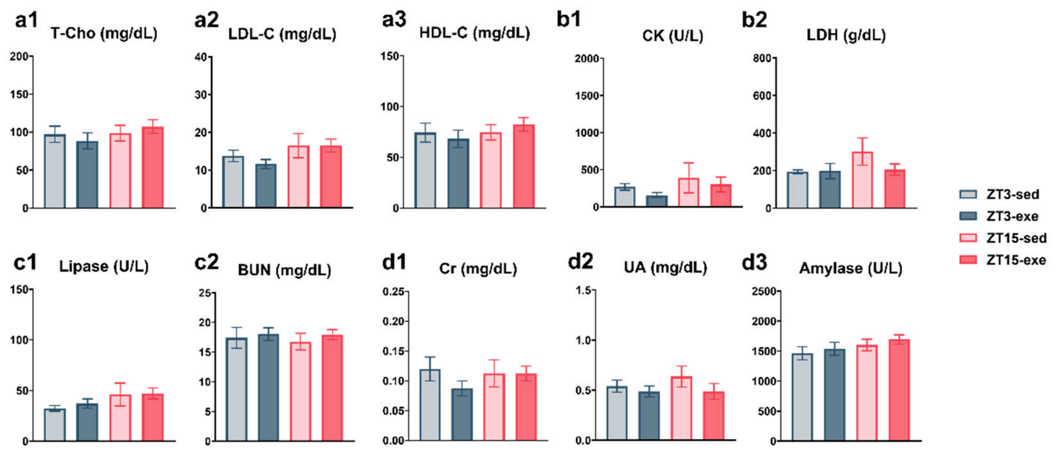

Figure S2. Plasma biochemical parameters in mice.

(a) Lipid metabolism: (a1) Total cholesterol (T-Cho, mg/dL); (a2) low-density lipoprotein cholesterol (LDL-C, mg/dL); (a3) high-density lipoprotein cholesterol (HDL-C, mg/dL). (b) Myocardial/tissue damage: (b1) Creatine kinase (CK, U/L); (b2) Lactate dehydrogenase (LDH, g/dL). (c) Pancreatic function: (c1) Amylase (U/L); (c2) Lipase (U/L). (d) Renal function: (d1) Blood urea nitrogen (BUN, mg/dL); (d2) Creatinine (Cr, mg/dL); (d3) Uric acid (UA, mg/dL). Data are presented as mean ± SEM (ZT3-sed n = 5; ZT3-exe n = 8; ZT15-sed n = 8; ZT15-exe n = 8). Statistical analysis was performed using two-way ANOVA (phase × exercise).

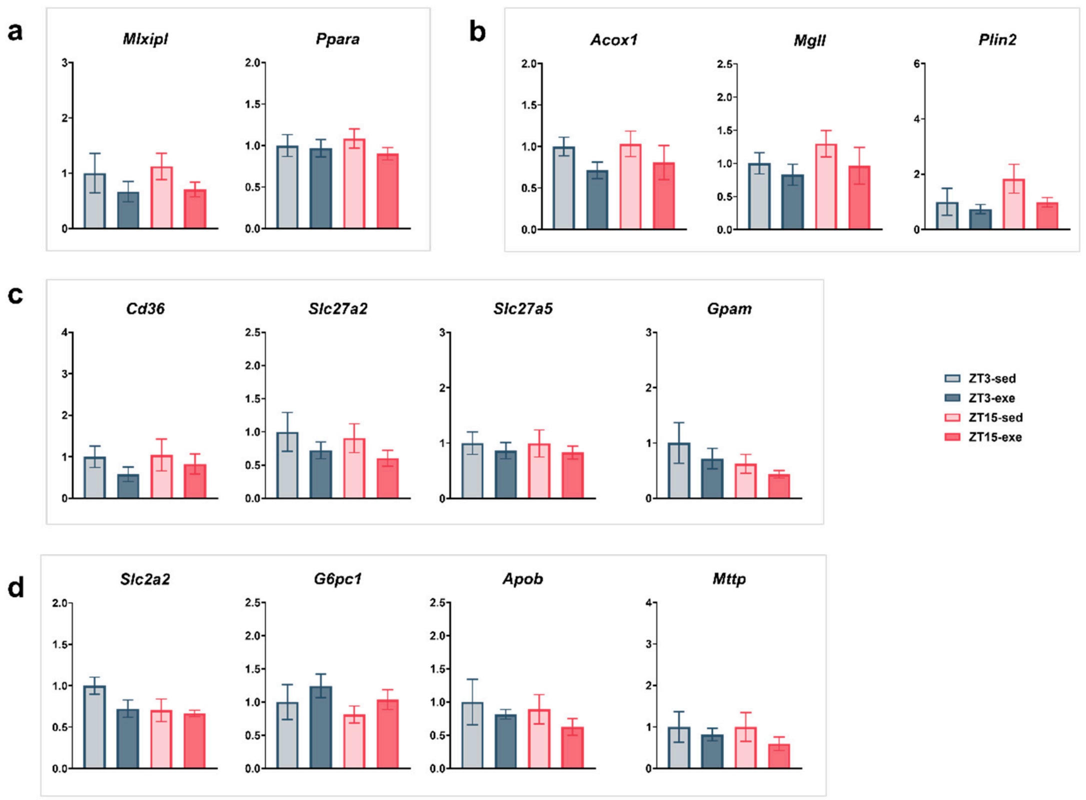

Figure S3. Effects of exercise timing and intervention on the expression of metabolism-related genes in mouse liver.

(a) Lipid synthesis and transcriptional control: *Mlxipl*, *Ppara*. (b) Lipid mobilization and oxidation: *Acox1*, *Mgll*, *Plin2*. (c) Fatty-acid transport and glycerolipid synthesis: *Cd36*, *Slc27a2*, *Slc27a5*, and *Gpam*. (d) Glucose metabolism and lipoprotein assembly: *Slc2a2*, *G6pc1*, *Apob*, and *Mttp*. Gene expression levels are shown as relative values normalized to reference genes and expressed as mean ± SEM (ZT3-sed n = 5, ZT3-exe n = 8, ZT15-sed n = 8, ZT15-exe n = 8). Statistical analysis was performed using two-way ANOVA (phase × exercise).
